# Gal3‐CaN‐Smurf1 Complex Sequestrates FLCN‐FNIPs to Facilitate TFEB Activation in Response to Endomembrane Damage

**DOI:** 10.1002/advs.202413241

**Published:** 2025-09-17

**Authors:** Qin Xia, Ziwan Liu, Gaoqing Feng, Wanting Xu, HuaHua Wang, Haihang Su, Jiaqian Li, Dan Liu, Jun Qu, Tonghui Yu, Lei Dong

**Affiliations:** ^1^ State Key Laboratory of Hearing and Balance Science and Key Laboratory of Molecular Medicine and Biological Diagnosis and Treatment (Ministry of Industry and Information Technology) Aerospace Center Hospital Advanced Technology Research Institute Tangshan Research Institute School of Life Science Beijing Institute of Technology Beijing 100081 China; ^2^ Department of General Surgery Aerospace Center Hospital Beijing 100049 China; ^3^ School of Physical and Mathematical Sciences Nanyang Technological University 639798 Singapore Singapore

**Keywords:** endomembrane damage, FLCN‐FNIPs, Gal3‐CaN‐Smurf1 complex, lysosomal stress, TFEB, ubiquitylation

## Abstract

Smurf1 mediates lysosomal biogenesis upon endomembrane damage by interacting with lysosomal injury sensor Gal3 and phosphatase CaN to form Gal3‐CaN‐Smurf1 complex, which is critical for TFEB dephosphorylation. However, whether Smurf1 plays a role in the inhibition of mTOR‐mediated TFEB phosphorylation is still unclear. TFEB phosphorylation by mTORC1 is strictly dependent on RagC/D GTPase activating protein FLCN. Here, we found that Smurf1 promotes the dissociation of RagC from TFEB upon lysosomal damage, selectively impairing TFEB phosphorylation. These findings suggest that the lysosomal damage‐induced Gal3‐CaN‐Smurf1 complex sequesters FLCN‐FNIPs to facilitate TFEB activation. This disruption of FLCN GAP function toward RagC/D impairs TFEB's lysosomal localization and phosphorylation. Notably, FLCN^K462R^ and/or FNIP2^K466R^ mutations reduce their binding affinity with the Gal3‐CaN‐Smurf1 complex, suggesting Smurf1‐mediated poly‐ubiquitylation of FLCN^K462^ and FNIP2^K466^ plays a role for pentamer formation. Indeed, sequestration of FLCN‐FNIPs stabilizes the Gal3‐CaN‐Smurf1 complex, wherein Smurf1 directly binds and ubiquitinates TFEB. This facilitates TFEB's dephosphorylation and activation. These findings indicate that Gal3‐CaN‐Smurf1 complex interconnects with the FLCN‐FNIPs to orchestrate TFEB localization and activity in response to lysosomal damage stress. Understanding Smurf1's regulation in the mTOR‐TFEB axis, which balances tumor growth and stress‐induced cell homeostasis, may provide novel therapeutic targets for tumor progression and drug resistance.

## Graphical abstract

The schematic diagram illustrates the role of the recruitment of Gal3‐CaN‐Smurf1 complex in maintaining lysosomal membrane homeostasis upon damage. On the one hand, the Gal3‐CaN‐Smurf1 complex sequesters FLCN‐FNIPs to block the activation of RagC, where the active form RagC‐GDP is responsible for TFEB phosphorylation. On the other hand, FLCN‐FNIPs stabilizes Gal3‐CaN‐Smurf1 complex for the recruitment and CaN‐mediated dephosphorylation of TFEB.

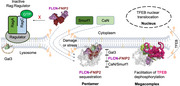

## 1 Introduction

The transcription factor EB (TFEB) is a master regulator for lysosomal biogenesis and autophagic degradation, which plays a crucial role in maintaining cellular homeostasis in adapting various stresses.^[^ ^1^, ^2^ ^]^ We generally believe that TFEB phosphorylation (mediate the retention of TFEB in the cytoplasm by binding 14‐3‐3) is the fundamental regulation for TFEB nuclear translocation. To begin with, previous reports showed that calcium release from lysosomes induces TFEB nuclear localization through the activation of the phosphatase calcineurin, which might be the common pathway activated by starvation,^[^ ^3^ ^]^ and LLOMe (L‐leucyl‐L‐leucine methyl ester).^[^ ^4^, ^5^ ^]^ Additionally, TFEB activity is prohibited by the major kinase complex mTORC1, which phosphorylates TFEB (S211) and keeps TFEB inactive in the cytoplasm by promoting the binding of TFEB with the cytosolic chaperone 14‐3‐3.^[^ ^6^, ^7^ ^]^ Of note, other kinases (MAPK, GSK3β) were also reported to participate in TFEB phosphorylation. Phosphorylation of TFEB S142 by MAPK contributes to TFEB cytosolic retention.^[^ ^7^ ^]^ GSK3β phosphorylates TFEB at S134 and S138 to promote the dynamic localization of TFEB on lysosomes, which making TFEB more accessible to lysosomal mTORC1.^[^ ^8^ ^]^ The lysosomal surface restores a signaling platform for the activation of mTORC1, which coordinates growth processes (phosphorylation of S6K and 4EBP1) in the response to nutrient availability.^[^ ^9^ ^]^ However, lysosomal damage selectively impairs the substrate‐specific mTORC1‐mediated phosphorylation of TFEB in a Rag GTPase‐dependent manner.^[^ ^10^, ^11^ ^]^ The tumor suppressor folliculin (FLCN, RagC and RagD activator) facilitates the transition of the Rag‐Ragulator complex (Rags encoded by four Rag genes, RagA or RagB forms heterodimers with RagC or RagD) from an inactive lysosomal FLCN complex (LFC) stable state to active released LFC form. FLCN promotes its guanosine triphosphatase (GTPase) activating protein (GAP) activity toward the GTPase RagC, which is strictly required for the activation of the RagC‐GDP.^[^ ^1^, ^12^ ^]^ FLCN is sequestered by the Rag‐Ragulator complex to facilitate mTOR activation. Loss‐of‐function FLCN mutation results in Birt‐Hogg‐Dubé syndrome through hyperactivation of TFEB nuclear transloation, characterized by benign skin tumours.^[^ ^2^ ^]^ However, the regulation of Rag‐Ragulator complex, which is responsible for mTOR activation, is regulated in the context of lysosomal damage is still unclear.

We noticed that LLOMe induced TFEB activation shares different mechanism from AA starvation induced TFEB activation. Previously, lysosomal membrane permeability (LMP), a hallmark of lysosomal damage, induces the formation of endosomal sorting complexes required for transport (ESCRT) for the lysosomal repair.^[^ ^13^ ^]^Gal3, acts as lysosomal damage sensor, detects and binds to exposed glycans on the luminal side of the lysosome after membrane rupture. Gal3 plays a key role in recruitment of ESCRT component ALIX (ALG‐2 Interacting Protein X), and ESCRT responses in a Ca^2+^ dependent manner at the early time point after lysosomal damage. On the other hand, those lysosomes with severe damage, for which ESCRT and PITT (phosphoinositide‐initiated membrane tethering and lipid transport pathway) are not sufficient to repair, are choose for autophagic degradation. This process mediated TFEB nuclear translocation is crucial for both clearance of damaged lysosomes and subsequent biogenesis through activation of the transcriptional program of autophagy/lysosome genes. To begin with, we previously identified Smurf1 (SMAD Specific E3 Ubiquitin Protein Ligase 1) involved in LLOMe mediated the damage response for lysosomal biogenesis by interacting with Gal3 and phosphatase calcineurin (PPP3CB).^[^ ^14^ ^]^ Gal3‐CaN‐Smurf1 complex dephosphorylates TFEB to promote its nuclear translocation in response to LLOMe.^[^ ^4^ ^]^ Additionally, the lipidation form of LC3 (LC3‐II) interacts with MCOLN1 (Mucolipin 1), further facilitating large amounts of calcium efflux that is essential for TFEB activation.^[^ ^10^ ^]^ We also found that MCOLN1‐mediated Ca^2+^ release is also required for activation of Smurf1‐CaN‐pathway. Notably, the knockdown of PPP3CB and/or PPP3CA, components of CaN, do not largely abolish TFEB activation induced by lysosomal damage, indicating that other TFEB phosphatases (activation) or kinase (inactivation) are involved in activating TFEB.^[^ ^10^ ^]^ Indeed, LLOMe partially impairs mTORC1 activity, indicated by the decreased phosphorylation of its selectively substrate TFEB,^[^ ^4^, ^10^ ^]^ suggesting that non‐canonical mTORC1 signaling is involved during lysosomal damage.^[^ ^15^ ^]^ Jia et al. reported a galectin‐based signal‐transduction system intersects with the regulators of mTOR on lysosomes and inhibits mTOR in response to LLOMe treatment.^[^ ^13^ ^]^ Galectin‐8 (Gal8) plays an indispensable role in transmitting lysosome damage signals by identifying exposed luminal glycans to effectively inactive of mTORC1.^[^ ^11^ ^]^ Further studies have shown that lysosomal damage selectively impairs the mTORC1‐mediated phosphorylation of TFEB in a Rag GTPase‐ and ATG conjugation system‐dependent manner dependent manner, leading to TFEB activation and subsequent lysosomal repair and clearance of damaged organelles.^[^ ^10^ ^]^ In summary, LLOMe induces the activation of TFEB via Gal3‐CaN‐Smurf1 complex mediated TFEB dephosphorylation. However, it is still unclear whether and how Gal3‐CaN‐Smurf1 complex mediates mTOR inactivation in response to lysosomal damage.

One critical issue is how lysosomal damaged signal prohibits TFEB phosphorylation mediated by mTOR. This promotes us to answer whether the Gal3‐CaN‐Smurf1 conjugation contributes to the uncoupling of TFEB from mTOR inactivation. This study identifies Smurf1‐mediated TFEB nuclear translocation requires RagC inactivation and promotes the dissociation of RagC from TFEB. We further dissect the molecular interactions and structural basis underlying the Gal3‐CaN‐Smurf1 mediated sequestration of FLCN‐FNIPs and subsequent TFEB activation in response to endomembrane damage. Our findings suggest that FLCN‐FNIPs interconnect with the Gal3‐CaN‐Smurf1 complex to orchestrate TFEB localization and activity in response to lysosomal damage stress. This study provides novel insights into the mechanisms of TFEB regulation and its potential therapeutic implications for tumor adaptive progression.

## 2 Result

### 2.1 Smurf1‐Mediated TFEB Nuclear Translocation in Response to Lysosomal Damage is Dependent on RagC Inactivation

**Figure 1:**
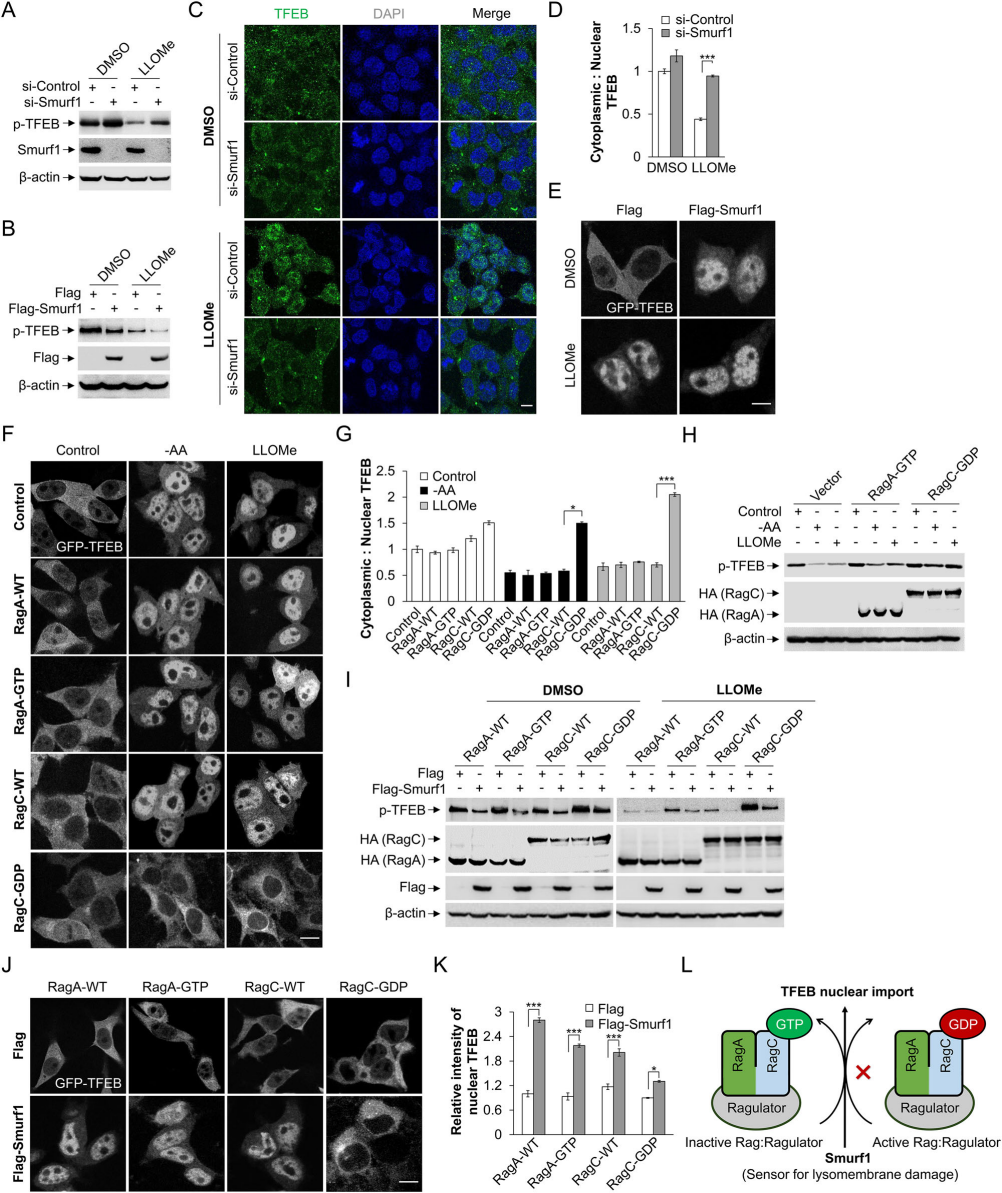
Smurf1 mediated TFEB nuclear translocation in response to lysosomal damage is dependent on RagC inactivation. A) HEK293 cells were transfected with Smurf1 or scramble siRNA oligos for 72 h and then treated with LLOMe (1 mM, 2 h) or equal volume DMSO. Cells were subjected to western blotting using p‐TFEB, Smurf1 and β‐actin antibodies. B) HEK293 cells were transfected with Flag or Flag‐Smurf1 for 24 h, and then were treated with LLOMe (1 mM, 2 h) or equal volume DMSO. Cells were subjected to western blotting using p‐TFEB, Flag and β‐actin antibodies. C,D) HEK293 cells were transfected with Smurf1 or scramble siRNA oligos for 72 h. Cells were treated with LLOMe (1 mM, 2 h) and equal volume DMSO before fixation and staining with TFEB antibody and DAPI (C). The ratio of cells with cytoplasmic to nuclear TFEB is shown in (D). E) HEK293 cells were transfected with Flag or Flag‐Smurf1 and GFP‐TFEB for 24 h. Cells were treated with LLOMe (1 mM, 2 h) or equal volume DMSO before fixation. F,G) HEK293 cells were transfected with control HA‐GST‐RagA‐WT, HA‐GST‐RagA‐GTP, HA‐GST‐RagC‐WT or HA‐GST‐RagC‐GDP and GFP‐TFEB for 24 h. Cells were treated with complete medium lacking AA or LLOMe (1 mM, 2 h) before fixation (F). The ratio of cells with cytoplasmic to nuclear TFEB is shown in (G). H) HEK293 cells were transfected with control or HA‐GST‐RagA‐GTP or HA‐GST‐RagC‐GDP for 24 h. Cells were treated with complete medium, lacking AA or LLOMe (1 mM, 2 h), and then cells were subjected to western blotting using p‐TFEB, HA and β‐actin antibodies. I) HEK293 cells were transfected with HA‐GST‐RagA‐WT, HA‐GST‐RagA‐GTP, HA‐GST‐RagC‐WT or HA‐GST‐RagC‐GDP and Flag or Flag‐Smurf1 for 24 h. Cells were treated with LLOMe (1 mM, 2 h) or equal volume DMSO, and then subjected to western blotting using p‐TFEB, HA, Flag, and β‐actin antibodies. J,K) GFP‐TFEB overexpressed HEK293 cells were transfected with HA‐GST‐RagA‐WT, HA‐GST‐RagA‐GTP, HA‐GST‐RagC‐WT or HA‐GST‐RagC‐GDP and Flag or Flag‐Smurf1 for 24 h before fixation (J). The relative intensity of TFEB in nucleus is shown in (K). L) Schematic diagram of Smurf1 promoting TFEB nuclear translocation under lysosomal damage dependent on RagC inactivation. Scale bar: 5 µm. *n* ≥ 50 cells per group. Data are representative of three independent experiments with three biological replicates. Results are presented as mean ± SD, *p < 0.05, ***p < 0.001 by two‐sided Student's *t*‐test.

Previously, Smurf1 interacts with endomembrane damage sensor Gal3 by bridging environmental stress with the core autophagosomal and autolysosomal machinery.^[^ ^4^, ^10^, ^16^, ^17^ ^]^ To identify if Smurf1 knockdown mitigates TFEB phosphorylation, cells were transfected with si‐Smurf1 or si‐control. We found that Smurf1 facilitates TFEB dephosphorylation in response to endomembrane damage, as evidenced by Smurf1 knocking down enhanced, but Smurf1 overexpression decreased, TFEB phosphorylation in different HEK293, LN229 and U343 cell lines (**Figure** **1**A,B; Figure S1A–D, Supporting Information). Consistently, fluorescence microscopy reveals that knocking down of Smurf1 impedes (Figure 1C,D), but overexpression of Smurf1 promotes (Figure 1E), TFEB nuclear translocation in response to endomembrane damage. Our previously study showed that formation of Smurf1‐PPP3CB‐Gal3 complex enhances dephosphorylation of transcription factor EB (TFEB) for nuclear translocation.^[^ ^4^ ^]^ However, whether Smurf1 plays a role in the mTOR‐mediated inhibition of TFEB phosphorylation for its nuclear translocation is still unclear.

Nutrition‐mediated mTORC1 activation promotes TFEB phosphorylation, which retains TFEB in the cytoplasm by promoting its interaction with 14‐3‐3 proteins.^[^ ^7^, ^18^ ^]^ The active form of RagC‐GDP (the component of Rag‐Ragulator complex) is required for the mTORC1‐mediated TFEB phosphorylation.^[^ ^2^, ^19^ ^]^ To identify whether Smurf1‐mediated TFEB translocation is partially dependent on RagC inactivation, we first overexpression of either RagA‐GTP or RagC‐GDP and treated cells with LLOMe. Strikingly, RagC‐GDP overexpression prohibits TFEB nuclear translocation (Figure 1F,G). Indeed, western blot analysis verify the RagC‐GDP overexpression enhance TFEB phosphorylation (Figure 1H). Of note, overexpression of RagC‐GDP blocks the effect of Smurf1‐mediated TFEB dephosphorylation and nuclear import (Figure 1I–K), suggesting Smurf1‐mediated TFEB nuclear translocation in response to lysosomal damage is at least partially dependent on RagC inactivation (Figure 1L).

### 2.2 Smurf1 Promotes the Disassociation of RagC from TFEB

**Figure 2:**
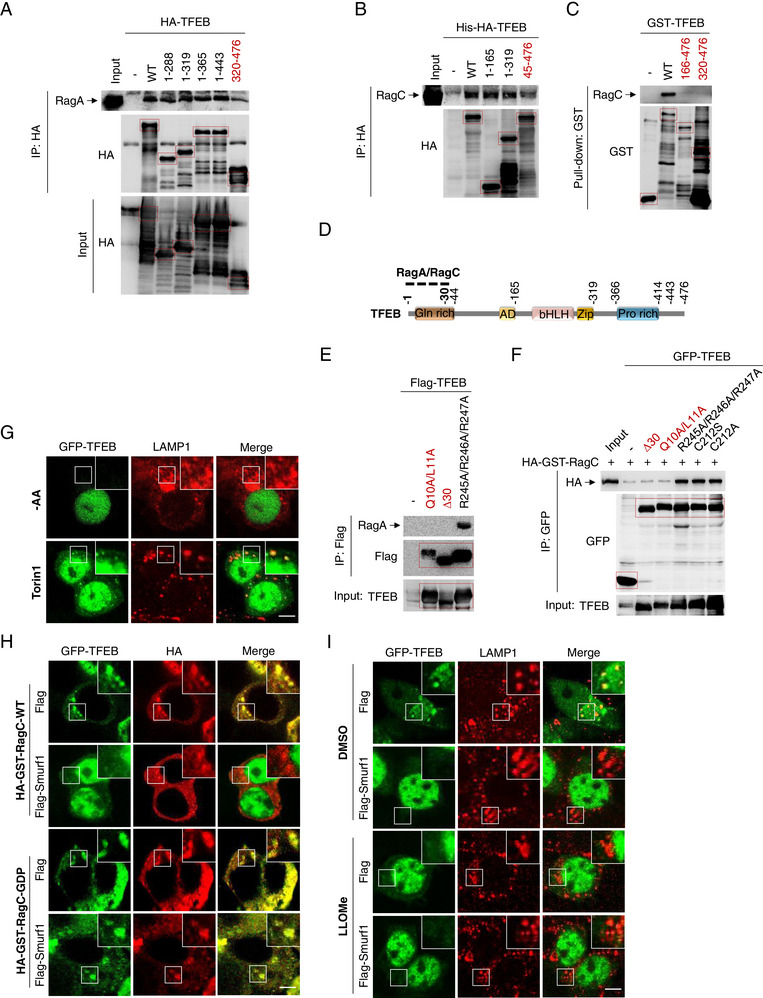
Smurf1 promotes the disassociation of RagC from TFEB. A) Co‐IP analysis of the interaction between HA‐TFEB constructs and endogenous RagA expressed in HEK293 cells. B) Co‐IP analysis of the interaction between His‐HA‐TFEB constructs purified from E. coli and endogenous RagC in HEK293 cells. C) GST Pull‐down analysis of the interaction between GST‐TFEB constructs purified from E. coli and endogenous RagC in HEK293 cells. D) Schematic diagram of mapping the interaction between RagA/RagC and TFEB. E) Co‐IP analysis of the interaction between Flag‐TFEB constructs and endogenous RagA expressed in HEK293 cells. F) Co‐IP analysis of the interaction between GFP‐TFEB constructs and HA‐GST‐RagC expressed in HEK293 cells. G) HEK293 cells were transfected with GFP‐TFEB for 24 h. Cells were treated with complete medium lacking AA or Torin1 (250 nM, 1 h) before fixation and staining with LAMP1 antibody. H) HEK293 cells were transfected with indicated plasmids for 24 h before fixation and staining with HA antibody. I) HEK293 cells were transfected with GFP‐TFEB and Flag or Flag‐Smurf1 for 24 h. Cells were treated with LLOMe (1 mM, 2 h) or equal volume DMSO before fixation and staining with LAMP1 antibody. Scale bar: 5 µm. *n* ≥ 50 cells per group. Data were representative of three independent experiments. Data are representative of three independent experiments with three biological replicates.

Previously, TFEB was identified to interact with Rag‐Ragulator complex (RagC) for its phosphorylation.^[^ ^19^, ^20^ ^]^ Next, to delineate the interaction domain of TFEB with the Rag‐Ragulator complex, we engineered a series of truncated TFEB constructs (1‐165, 1–250, 1–288, 1–319, 1–365, 1–443, 45–476, 166–476, 251–476, 320–476). The binding affinity of TFEB N‐terminal region was determined to interact with RagA, as constructs 1–250, 1–288, 1–319, 1–365, and 1–443 associated with RagA, whereas constructs 251–476, 289–476 and 320–476 did not (**Figure** **2**A; Figure S1E, Supporting Information). Similarly, TFEB constructs 1–165, 1–250, 1–319, 1–365, 1–443, but not constructs 45–476, 166–476, 251–476 and 320–476, binds to RagC (Figure 2B,C; Figure S1F,G, Supporting Information). These data collectively suggest that the N‐terminal domain of TFEB, specifically residues 1–44, is requisite for the engagement with the Rag‐Ragulator complex. We further identified TFEB‐Δ30 and TFEB‐Q10A/L11A mutation significantly reduced its binding affinity with RagA and RagC (Figure 2D–F).

Activated RagC GDP‐bound formed by FLCN GAP activity physically recruits TFEB to lysosome surface, thereby promoting its phosphorylation by mTORC1; phosphorylated TFEB is then bounded to 14‐3‐3 and sequestered in the cytoplasm, thus suppressing TFEB activation and translocation into the nucleus.^[^ ^2^ ^]^ Similar findings were recently reported for the regulation of TFE3.^[^ ^21^ ^]^ To elucidate the subcellular (nucleus/cytoplasm) distribution of TFEB in the context of AA deficiency and inhibition of mTORC1, we overexpressed TFEB and analyzed the immunofluorescent staining. Consistently, Torin1 treatment markedly induced the localization of TFEB to the lysosomal membrane, in contrast to the nuclear translocation prompted by amino acid nutrient deprivation without lysosomal retention (Figure 2G). Notably, the overexpression of the constitutively active RagC‐GDP variant significantly impeded Smurf1‐mediated nuclear translocation of TFEB (Figure 2H), suggesting that the disassociation of TFEB from Rag‐Ragulator complex (RagC inactive form) is required for Smurf1‐mediated TFEB nuclear translocation. Consistently, treatment with LLOMe facilitated the nuclear import of TFEB without lysosomal sequestration (Figure 2I). We may wonder whether and how Smurf1 inactives RagC in response to lysosomal damage.

### 2.3 Gal3 Triggers the Sequestration of FLCN‐FNIPs in Response to LLOMe

**Figure 3:**
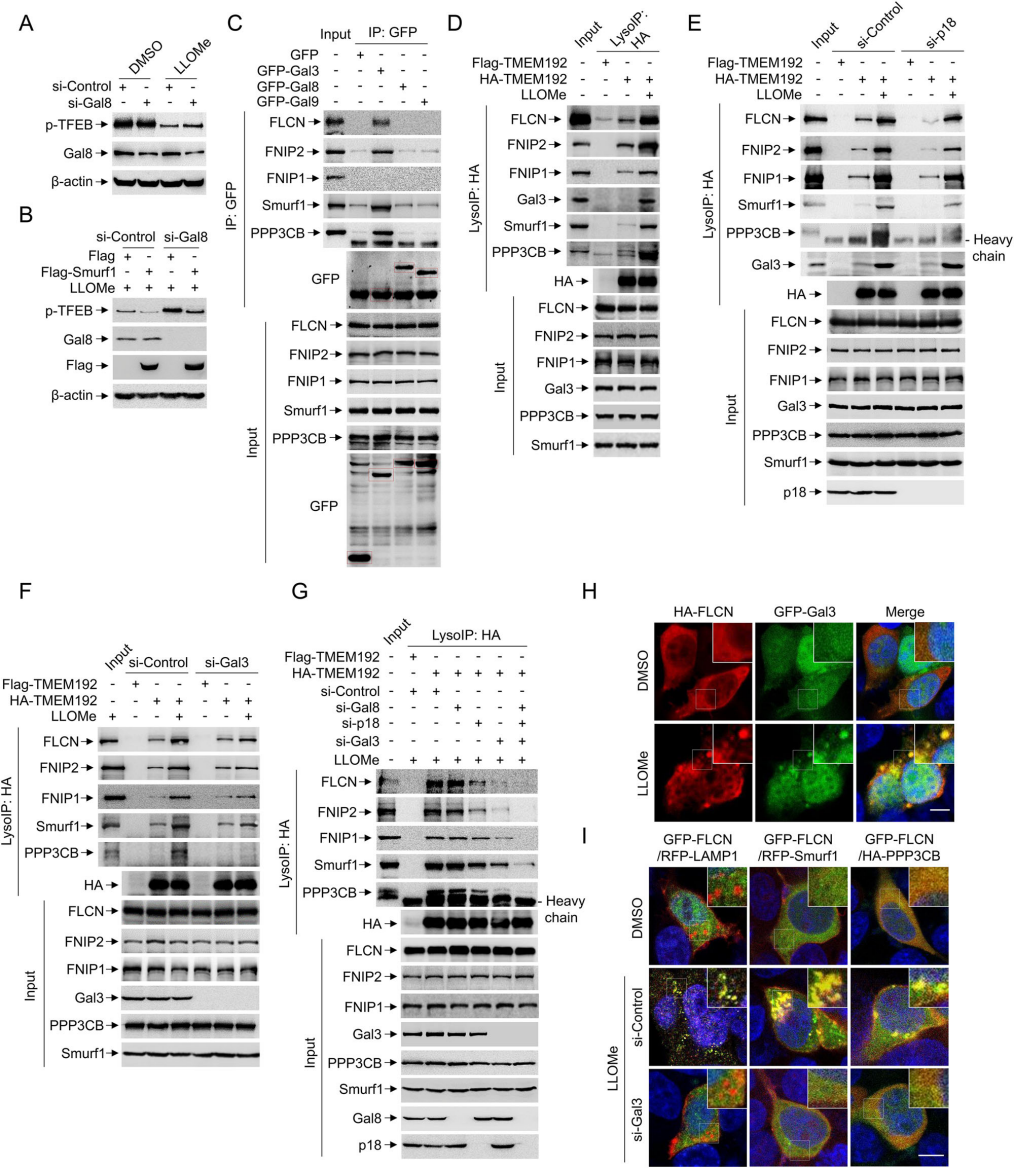
Gal3 triggers the sequestration of FLCN‐FNIPs in response to LLOMe. A) HEK293 cells were transfected with Gal8 or scramble siRNA oligos for 72 h and treated with LLOMe (1 mM, 2 h) or equal volume DMSO. Cells were subjected to western blotting using p‐TFEB, Gal8 and β‐actin antibodies. B) HEK293 cells were transfected with Gal8 or scramble siRNA oligos for 48 h and transfected with Flag or Flag‐Smurf1 for another 24 h. Cells were treated with LLOMe (1 mM, 2 h) and subjected to western blotting using p‐TFEB, Gal8, Flag and β‐actin antibodies. C) HEK293 cells were transfected with GFP, GFP‐Gal3, GFP‐Gal8 or GFP‐Gal9 for 24 h and then IP with GFP for immunoblotting with indicated antibodies. D) HEK293 cells were transfected with HA‐TMEM192 or Flag‐TMEM192 for 24 h and treated with LLOMe (1 mM, 2 h). Cell lysates were then LysoIP with HA for immunoblotting with indicated antibodies. E,F) HEK293 cells were transfected with pl8 (E) or Gal3 (F) or scramble siRNA oligos for 48 h and transfected with HA‐TMEM192 or Flag‐TMEM192 for another 24 h. Cells were treated with LLOMe (1 mM, 2 h) and subjected to LysoIP with HA for immunoblotting with indicated antibodies. G) HEK293 cells were transfected with pl8, Gal3 or Gal8 or scramble siRNA oligos for 48 h and transfected with HA‐TMEM192 or Flag‐TMEM192 for another 24 h. Cells were treated with LLOMe (1 mM, 2 h) or equal volume DMSO and subjected to LysoIP with HA for immunoblotting with indicated antibodies. H) HEK293 cells were transfected with HA‐FLCN and GFP‐Gal3 for 24 h. Cells were treated with or without LLOMe (1 mM, 2 h) and fixed and stained with HA antibody and DAPI. I) HEK293 cells were transfected with Gal3 or scramble siRNA oligos for 48 h and transfected with indicated plasmids for another 24 h. Cells were treated with or without LLOMe (1 mM, 2 h) and fixed and stained with DAPI. Scale bar: 5 µm. *n* ≥ 50 cells per group. Data are representative of three independent experiments with three biological replicates.

Previous studies showed that lysosomal damage marker Gal8 mediates the mTORC1 inactivation upon lysosome damage.^[^ ^11^ ^]^ Consistently, si‐Gal8 enhanced TFEB phosphorylation upon LLOMe treatment (**Figure** **3**A). However, Smurf1 overexpression restored TFEB dephosphorylation even under si‐Gal8 conditions, indicating that Gal8 is not required for Smurf1‐mediated mTOR inhibition in response to LLOMe (Figure 3B). Subsequent screening of lysosomal damage related galectins revealed that Gal3, but not Gal8 or Gal9, has the capacity to sequester FLCN‐FNIPs (Figure 3C). Previously, Gal3 detects and binds to exposed glycans on the luminal side of the lysosome in response to severe membrane rupture.^[^ ^11^, ^13^, ^16^ ^]^ We found that Gal3 recruits both PPP3CB and Smurf1 to form Gal3‐CaN‐Smurf1 complex for assistance of TFEB recruitment and conformational correction to facilitate its dephosphorylation.^[^ ^4^ ^]^ Next, to test whether Gal3‐CaN‐Smurf1 complex sequesters the “switch” (FLCN‐FNIPs) for potentially inhibition of TFEB lysosomal localization to mTORC1 Rag‐Ragulator in response to lysosomal membrane damage, we first utilize transmembrane protein 192 (TMEM192), which retains lysosomal localization upon overexpression,^[^ ^13^, ^22^ ^]^ for lysosome immunoprecipitation (LysoIP) to identify lysosomal proteins in both with and without lysosomal damage conditions. LysoIP identified the significant recruitment of the all the components of both the FLCN‐FNIPs and Gal3‐CaN‐Smurf1 complexes to the lysosomal membrane in response to LLOMe compared to control, suggesting FLCN‐FNIPs sequestration in the lysosomal membrane upon lysosomal damage(Figure 3D). We generally believe that under normal conditions, FLCN (RagC and RagD activator) is sequestered outside the lysosomal membrane by Rag‐Ragulator complex, and loss of function FLCN mutation leads to Birt‐Hogg‐Dubé syndrome, a disorder characterized by benign skin tumors.^[^ ^2^ ^]^ We then hypothesis that FLCN is sequestered by the Gal3‐CaN‐Smurf1 complex in order to keep it away from the Rag‐Ragulator complex, preventing FLCN from exerting its GAP enzyme activity for RagC and RagD activation. Indeed, we found the sequestration of FLCN‐FNIPs at the lysosomal membrane was partially dependent on the Rag‐Ragulator complex, as evidenced by the binding affinity of FLCN‐FNIPs with lysosomal membrane is slightly disrupted by si‐p18(a component of the Rag‐Ragulator complex) in response to LLOMe (Figure 3E). Notably, knocking down Gal3 significantly blocked the retention of FLCN‐FNIPs, CaN, and Smurf1 with TMEM192, indicating that Gal3 is required for the sequestration of FLCN‐FNIPs at the lysosomal membrane in response to LLOMe (Figure 3F). This notion is consistent across LN229 and U343 GBM cell lines that knocking down Gal3 significantly blocked the sequestration of FLCN‐FNIPs at the lysosomal membrane in response to LLOMe (Figure S1H,I, Supporting Information). We previously identified Gal3 is also required for the recruitment of CaN, and Smurf1 in lysosomal damage.^[^ ^4^ ^]^ LysoIP revealed that Gal3‐CaN‐Smurf1 complexes significantly sequestered FLCN‐FNIPs in response to lysosomal damage, as evidenced by the knockdown of Gal3 and/or Gal3 together with pl8 and Gal8 markedly reduced FLCN‐FNIPs lysosomal membrane localization compared to si‐p18 and/or si‐Gal8 alone under LLOMe treatment (Figure 3G). Fluorescence staining confirmed the colocalization of HA‐FLCN and GFP‐Gal3 following LLOMe treatment (Figure 3H). Consistently, Gal3 knockdown significantly decreased GFP‐FLCN colocalized with the lysosomal marker LAMP1, Smurf1 and PPP3CB in response to lysosomal damage (Figure 3I), suggesting that Gal3 plays an initial role in the sequestration of FLCN‐FNIPs in response to LLOMe.

### 2.4 Smurf1 Directly Interacts with and Ubiquitylates FLCN

**Figure 4:**
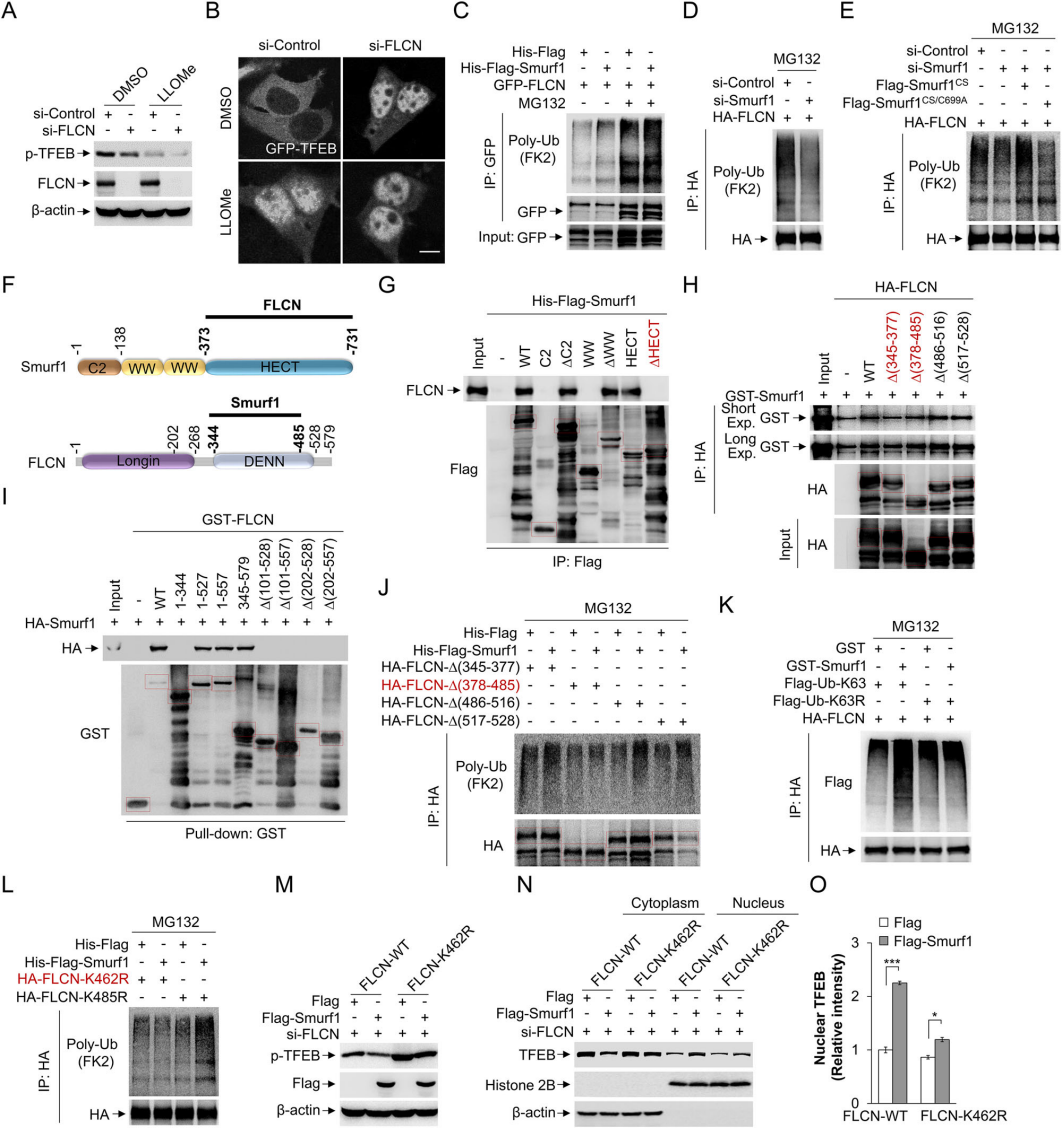
Smurf1 directly interacts with and ubiquitylates FLCN. A) HEK293 cells were transfected with FLCN or scramble siRNA oligos for 72 h, and then were treated with LLOMe (1 mM, 2 h) or equal volume DMSO. Cells were subjected to western blotting using p‐TFEB, FLCN and β‐actin antibodies. B) HEK293 cells were transfected with FLCN or scramble siRNA oligos for 48 h and transfected with GFP‐TFEB for another 24 h. Cells were treated with LLOMe (1 mM, 2 h) or equal volume DMSO before fixation. C) HEK293 cells were transfected with GFP‐FLCN for 12 h and treated with or without MG132 (10 µM) for another 12 h. Cell lysates were then incubated with or without His‐Flag‐Smurf1 and IP with GFP for immunoblotting with antibodies against Ub and GFP. D) HEK293 cells were transfected with Smurf1 or scramble siRNA oligos for 48 h and transfected with HA‐FLCN for another 12 h. Cells were then treated with treated with MG132 (10 µM) for 12 h. Cell lysates were then IP with HA for immunoblotting with antibodies against Ub and HA. E) HEK293 cells were transfected with Smurf1 or scramble siRNA oligos for 48 h and transfected with Flag or Flag‐Smurf1‐CS (Codon Switch: resistance to si‐Smurf1) or Flag‐Smurf1‐CS/C699A and HA‐FLCN for another 12 h. Cells were then treated with treated with MG132 (10 µM) for 12 h. Cell lysates were then IP with HA for immunoblotting with antibodies against Ub and HA. F) Schematic diagram of mapping the direct interaction between Smurf1 and FLCN. G) Co‐IP analysis of the interaction between endogenous FLCN in HEK293 cells and His‐Flag‐Smurf1 constructs purified from E. coli. H) Co‐IP analysis of the interaction between HA‐FLCN constructs expressed in HEK293 cells and GST‐Smurf1 purified from E. coli. I) GST Pull‐down analysis of the interaction between HA‐Smurf1 expressed in HEK293 cells and GST‐FLCN constructs purified from E. coli. J) HEK293 cells were transfected with HA‐FLCN constructs for 12 h and treated with MG132 (10 µM) for another 12 h. Cell lysates were then incubated with or without His‐Flag‐Smurf1 and IP with HA for immunoblotting with antibodies against Ub and HA. K) HEK293 cells were transfected with HA‐FLCN and Flag‐Ub‐K63 or Flag‐Ub‐K63R for 12 h and treated with MG132 (10 µM) for another 12 h. Cell lysates were then incubated with or without GST‐Smurf1 and IP with HA for immunoblotting with antibodies against Flag and HA. L) HEK293 cells were transfected with HA‐FLCN‐K462R or HA‐FLCN‐K485R for 12 h and treated with MG132 (10 µM) for another 12 h. Cell lysates were then incubated with or without His‐Flag‐Smurf1 and IP with HA for immunoblotting with antibodies against Ub and HA. M) HEK293 cells were transfected with FLCN siRNA oligos for 48 h and transfected with Flag or Flag‐Smurf1 and HA‐FLCN‐WT or HA‐FLCN‐K462R for another 12 h. Cells were subjected to western blotting using p‐TFEB, Flag and β‐actin antibodies. N,O) HEK293 cells were transfected with FLCN siRNA oligos for 48 h and transfected with Flag‐Smurf1 and HA‐FLCN‐WT or HA‐FLCN‐K462R for another 12 h. Cells were subjected to nuclear and cytoplasmic separation assay and western blotting analysis using anti‐TFEB, anti‐β‐actin and anti‐Histone 2B antibodies (N). The relative intensity of TFEB in nucleus is shown in (O). Scale bar: 5 µm. *n* ≥ 50 cells per group. Data are representative of three independent experiments with three biological replicates. Results are presented as mean ± SD, *p < 0.05, ***p < 0.001 by two‐sided Student's t‐test.

GABARAP sequestration of the FLCN‐FNIPs axis serves as the coordinator of lysosomal homeostasis with perturbations within the autophagy‐lysosomal network.^[^ ^12^, ^23^ ^]^ GABARAP directly sequestrates FLCN‐FNIP complex on intracellular membranes impairs the binding of cytosolic RagC‐GTP with mTORC1 substrate TFEB, thus reduces TFEB phosphorylation.^[^ ^23^ ^]^ To identify whether it is specificity on sequestration of FLCN‐FNIPs in regulation of mTORC1‐mediated TFEB phosphorylation in the context of lysosomal damage, we first knocked down FLCN and found si‐FLCN significantly enhanced the dephosphorylation and nuclear translocation of TFEB in response to LLOMe (**Figure** **4**A,B). Given Smurf1 is an E3 ligase, we investigated its influence on FLCN ubiquitination. We conducted ubiquitination assays utilizing GFP‐tagged FLCN (GFP‐FLCN) as a substrate in conjunction with His‐Flag‐tagged Smurf1 in the treatment of proteasomal inhibitor MG132. It shows that Smurf1 facilitates the ubiquitylation of immunoprecipitated GFP‐FLCN (Figure 4C). In contrast, Smurf1 knockdown attenuated FLCN ubiquitination, evidenced by siRNA against Smurf1 (si‐Smurf1) reducing polyubiquitination of HA‐tagged FLCN (Figure 4D). Additionally, to verify the ubiquitylation of FLCN is directly mediated by Smurf1, we supplemented si‐Smurf1 cells with Flag‐Smurf1‐CS (codon switch, resistant to si‐Smurf1) and its E3 ligase‐deficient mutant (C699A) in the treatment of MG132. Immunoprecipitation analysis showed that the reduction in ubiquitylated FLCN observed in si‐Smurf1 group could be restored by Flag‐Smurf1‐CS, but not by ligase‐deficient Flag‐Smurf1‐ CS^C699A^ mutant in HEK293, LN229, and U343 cell lines (Figure 4E; Figure S2A,B, Supporting Information), indicating Smurf1 directly mediates FLCN ubiquitylation. Next, to dissert the binding domain between Smurf1 and FLCN, we mapped the specific Smurf1 (containing C2, WW and HECT domain) and FLCN (containing Longin and DENN domain) by constructing truncated FLCN and Smurf1 as indicated in Figure 3F. We found Smurf1 HECT domain and FLCN DENN domain (345‐485) were required for their direct interaction *ex vivo* (Figure 4F–I; Figure S2C, Supporting Information).

Next, to determine Smurf1‐mediated FLCN ubiquitylation at which lysine (K) site, we performed ubiquitination assays using various FLCN constructs (1‐344, 1–528, 203–579, 345–579, ∆345‐377, ∆378‐485, ∆486‐516, ∆517‐528, ∆202‐344, ∆345‐528) as a substrate in the context of Smurf1 overexpression. Our results indicated that Smurf1 ubiquitinates the FLCN fragment between residues 378–485, as evidenced by the significant reduction in Smurf1‐mediated ubiquitination for the HA‐FLCN (Δ378‐485) construct, but not the constructs without the 378–485 fragment (Figure 4J; Figure S2D–F, Supporting Information). Additionally, we discovered that Smurf1 specifically mediates the conjugation of K63‐linked polyubiquitin to FLCN (Figure 4K; Figure S2G, Supporting Information). Consistently, the ubiquitination level of FLCN with a K48R mutation, but not wild‐type K48, was markedly enhanced by GST‐Smurf1, further indicating that Smurf1 primarily mediates K63‐linked ubiquitination of FLCN (Figure S2H, Supporting Information). To identify which specific lysine within the FLCN fragment 378–485 (K462 and K485) contributes to Smurf1‐mediated K63 ubiquitylation, we generated FLCN‐K462R and FLCN‐K485R mutants. Our findings showed that HA‐FLCN‐K462R, but not HA‐FLCN‐K485R, significantly impeded Smurf1‐mediated ubiquitination (Figure 4L). Notably, FLCN‐K462R mutant partially rescued the blockage of phosphorylation mediated by Flag‐Smurf1 overexpression (Figure 4M). The nuclear fission assays further confirmed the reduced nuclear import of TFEB by FLCN‐K462R (Figure 4N,O; Figure S2I, Supporting Information), indicating Smurf1‐mediated K63‐linked ubiquitylation of FLCN at K462 plays a promotive role in the sequestration of FLCN‐FNIP complex to inhibit mTORC1‐mediated TFEB dephosphorylation.

### 2.5 Gal3‐CaN‐Smurf1 Complex Interacts with FLCN

**Figure 5:**
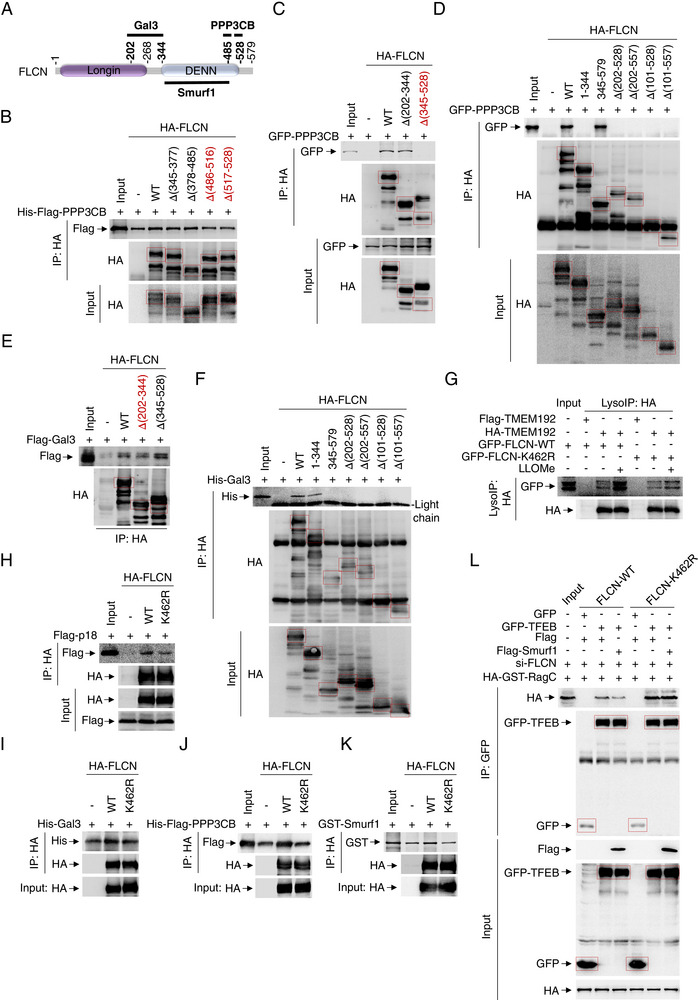
Gal3‐CaN‐Smurf1 complex interacts with FLCN. A) Schematic diagram of mapping the interaction between FLCN and Gal3‐PPP3CB‐Smurf1. B) Co‐IP analysis of the interaction between HA‐FLCN constructs expressed in HEK293 cells and His‐Flag‐PPP3CB purified from E. coli. C,D) Co‐IP analysis of the interaction between HA‐FLCN constructs and GFP‐PPP3CB expressed in HEK293 cells. E) Co‐IP analysis of the interaction between HA‐FLCN constructs and Flag‐Gal3 expressed in HEK293 cells. F) Co‐IP analysis of the interaction between HA‐FLCN constructs expressed in HEK293 cells and His‐Gal3 purified from E. coli. G) HEK293 cells were transfected with GFP‐FLCN‐WT or GFP‐FLCN‐K462R and HA‐TMEM192 or Flag‐TMEM192 for 22 h and treated with LLOMe (1 mM, 2 h) or equal volume DMSO. Cell lysates were then LysoIP with HA for immunoblotting with antibodies against HA and GFP. H) Co‐IP analysis of the interaction between HA‐FLCN constructs and Flag‐p18 expressed in HEK293 cells. I–K) Co‐IP analysis of the interaction between HA‐FLCN constructs expressed in HEK293 cells and His‐Gal3 (I) or His‐Flag‐PPP3CB (J) or GST‐Smurf1 (K) purified from E. coli. L) HEK293 cells were transfected with FLCN siRNA oligos for 48 h and transfected with indicated plasmids for another 24 h. Co‐IP analysis of the interaction between HA‐GST‐RagC and GFP‐TFEB expressed in HEK293 cells. Data are representative of three independent experiments with three biological replicates.

To elucidate whether the Gal3‐CaN‐Smurf1 complex, as the integrated component for TFEB dephosphorylation in response to lysosomal damage, also contributes to sequestration of FLCN, we conducted interaction assays. Surprisingly, we found that FLCN directly interacts with both Gal3 and Smurf1, and indirectly with PPP3CB (**Figure** **5**A). Specifically, using various FLCN constructs (1‐344, 345–579, Δ101‐528, Δ101‐557, Δ202‐528, Δ202‐557, Δ345‐377, Δ202‐344, Δ345‐528, Δ378‐485, Δ486‐516, Δ517‐528), we mapped the interaction regions and identified that PPP3CB interacts with the FLCN DENN (486‐528) region, as evidenced by significantly reduced interaction with PPP3CB in the Δ486‐516, Δ517‐528 and Δ345‐528 constructs (Figure 5B–D). Similarly, Gal3 was found to directly interact with the FLCN linker (202‐344) region, as the FLCN (Δ202‐344) significantly diminished its interaction with Gal3 (Figure 5E,F). Importantly, to determine the impact of the FLCN K462 mutation on lysosomal membrane interaction, we observed a decreased localization of the FLCN K462R mutant with the lysosomal membrane marker TMEM192 upon lysosome damage (Figure 5G). To dissect the interaction affinity of the FLCN K462 mutation with either the Rag‐Ragulator or the Gal3‐CaN‐Smurf1 complex, we performed immunoprecipitation assays. The FLCN K462R mutant showed reduced interaction with Flag‐p18 (Figure 5H) and the Gal3‐CaN‐Smurf1 complex (Figure 5I–K). Notably, Smurf1 overexpression decreased the interaction of GFP‐TFEB and HA‐GST‐RagC in wild‐type FLCN, but not in FLCN‐K462R mutant expressed cells (Figure 5L).

We have identified that the HECT domain of Smurf1 interacts with the linker regions of FNIP1 (residues 575–901) and FNIP2 (residues 529–790) (Figure S3A, Supporting Information). Initially, we observed that both FNIP1 (under LLOMe treatment; Figure S3B, S3>‐1A, Supporting Information) and FNIP2 (Figure S3C, Supporting Information) engage with Smurf1. To identify the precise interaction regions between FNIPs and Smurf1, we engineered a series of FNIP1 constructs (1‐574, 1–901, 272–1138, 575–901, 902–1138, 575–1138) and FNIP2 (1‐174, 1–528, 1–790, 529–1114, 791–1114, 392–1114, 1–941, 942–1114). Our mapping revealed that GST‐Smurf1 interacts with the HA‐FNIP1 linker region (575‐901) under LLOMe treatment, as demonstrated by the strong interactions with the HA‐FNIP1 in 575–901 constructs (Figure S3D, S3‐1B, Supporting Information). Similarly, GST‐Smurf1 was found to associate with the HA‐FNIP2 linker region (529‐1114) (Figure S3E, Supporting Information), whereas the interaction with the HA‐FNIP2 (791‐1114) was significantly diminished in Smurf1 interactions (Figures S3‐1C, D and S4F, Supporting Information). To further investigate whether Smurf1 mediates FNIPs ubiquitination and to identify the specific lysine (K) sites involved, we performed ubiquitination assays. The results indicated that Smurf1 does not mediate FNIP1 (Figure S3G, Supporting Information), but promotes FNIP2 ubiquitination in response to both LLOMe and MG132 (Figure S3H, S3‐1E, Supporting Information). Immunoprecipitation analysis indicated that the reduction in ubiquitylated FNIP2 observed in si‐Smurf1 condition could be restored by Flag‐Smurf1‐CS, but not by ligase‐deficient Flag‐Smurf1‐CS^C699A^ mutant (Figure S3‐1F, Supporting Information). We discovered that the ubiquitination level of FNIP2 with a K48R mutated ubiquitin, but not wild‐type K48, was markedly enhanced by GST‐Smurf1 (Figure S3I, Supporting Information). Consistently, FNIP2 linked K63 ubiquitin, but not K63R mutant ubiquitin, was markedly enhanced by GST‐Smurf1 (Figure S3J, Supporting Information), indicating that Smurf1 primarily mediates K63‐linked ubiquitination of FNIP2. We also conducted ubiquitination assays using various FNIP2 constructs (1‐174, 1–391, 1–528, 392–1114, 529–1114) as substrates in the context of Smurf1 overexpression. Our results indicated that Smurf1 ubiquitinates the FNIP2 fragment between residues 392–528, as indicated by the substantial increase in Smurf1‐mediated ubiquitination for the HA‐FNIP2 (392‐1114 and 1–528) constructs (Figure S3K,L, Supporting Information), but not the HA‐FNIP2 (1‐174, 1–391 and 529–1114) constructs (Figure S3K,L, S3‐1G, Supporting Information). Subsequently, we mutated all eight lysine sites within FNIP2 (392‐528), identified lysine 466 (HA‐FNIP2‐K466) as a critical site for Smurf1‐mediated ubiquitination (Figure S3M, S3‐1H–K, Supporting Information).

### 2.6 Gal3‐CaN‐Smurf1 Complex Facilitates FLCN‐FNIPs Sequestration

**Figure 6:**
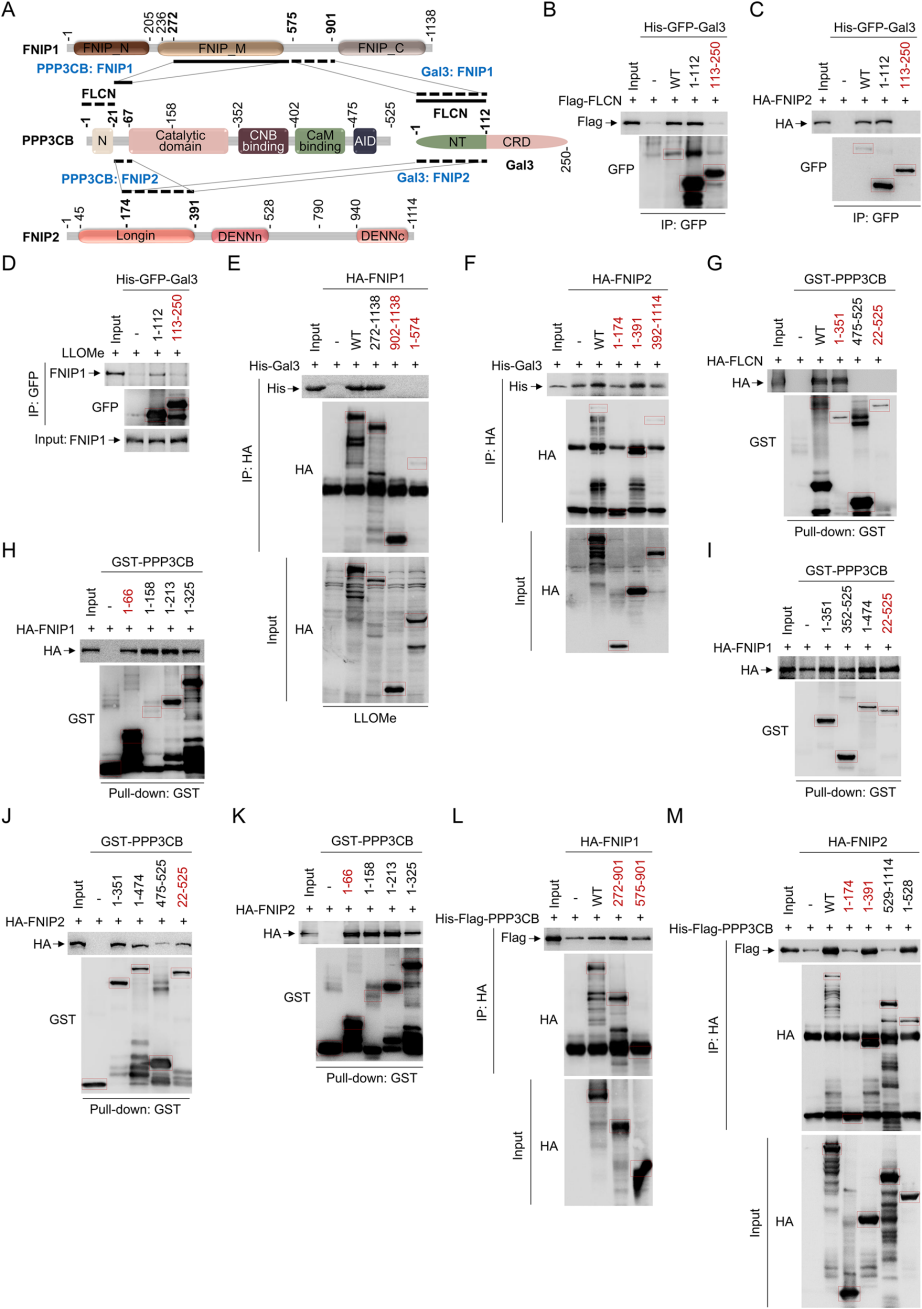
Gal3‐CaN‐Smurf1 complex facilitates FLCN‐FNIPs sequestration. A) Schematic diagram of mapping the interaction between PPP3CB or Gal3 and FNIP1 or FNIP2. B,C) Co‐IP analysis of the interaction between Flag‐FLCN (B) or HA‐FNIP2 (C) expressed in HEK293 cells and His‐GFP‐Gal3 constructs purified from E. coli. D) Co‐IP analysis of the interaction between His‐GFP‐Gal3 constructs purified from E. coli and endogenous FNIP1 in HEK293 cells treated with LLOMe (1 mM, 2 h). E,F) Co‐IP analysis of the interaction between HA‐FNIP1 (E) or HA‐FNIP2 (F) constructs expressed in HEK293 cells and His‐Gal3 purified from E. coli. G–K) GST Pull‐down analysis of the interaction between GST‐PPP3CB constructs purified from E. coli and HA‐FLCN (G), HA‐FNIP1 (H, I) or HA‐FNIP2 (J, K) expressed in HEK293 cells. L,M) Co‐IP analysis of the interaction between HA‐FNIP1 (L) or HA‐FNIP2 (M) constructs expressed in HEK293 cells and His‐Flag‐PPP3CB purified from E. coli. Data are representative of three independent experiments with three biological replicates.

To elucidate whether the Gal3‐CaN‐Smurf1 complex facilitates the sequestration of FLCN‐FNIPs, we conducted interaction assays and found that both PPP3CB and Gal3 interact with FNIPs (**Figure** **6**A). Specifically, we mapped the interaction regions and identified that FLCN, FNIP2, and FNIP1 (under LLOMe conditions) interact with the Gal3 NT (1‐112) domain (Figure 6B–D). Conversely, Gal3 was found to interact with the FNIP1 linker (575‐901) region in response to LLOMe, as FNIP1 (1‐574, 902–1138) significantly diminished its interaction with Gal3 (Figure 6E; Figure S4A,B, Supporting Information). Similarly, Gal3 interacts with the FNIP2 Longin (175‐391) region, as evidenced by the significant reduction in interaction with FNIP2 (1‐174), but not FNIP2 (1‐391) (Figure 6F; Figure S4C,D, Supporting Information). Additionally, FLCN interacts with the PPP3CB N‐terminal (1‐21) domain (Figure 6G; Figure S4E, Supporting Information). FNIP1 was found to interact with the PPP3CB linker (22‐66) region, as PPP3CB (1‐66) and PPP3CB (22‐525) significantly interact with FNIP1 (Figure 6H,I) and FNIP2 (Figure 6J,K). Conversely, PPP3CB interacts with the FNIP1 FNIP_M (272‐574) region, as evidenced by the significant reduction in its interaction with FNIP1 (575‐901), but not FNIP1 (272‐901) (Figure 6L; Figure S4F, Supporting Information). Similarly, PPP3CB interacts with the FNIP2 Longin (175‐391) region, as evidenced by the significant reduction in interaction with FNIP2 (1‐174), but not FNIP2 (1‐391) (Figure 6M; Figure S4G, Supporting Information).

### 2.7 Structure Basis for the Sequestration of the FLCN‐FNIPs by Gal3‐CaN‐Smurf1 Complex

**Figure 7:**
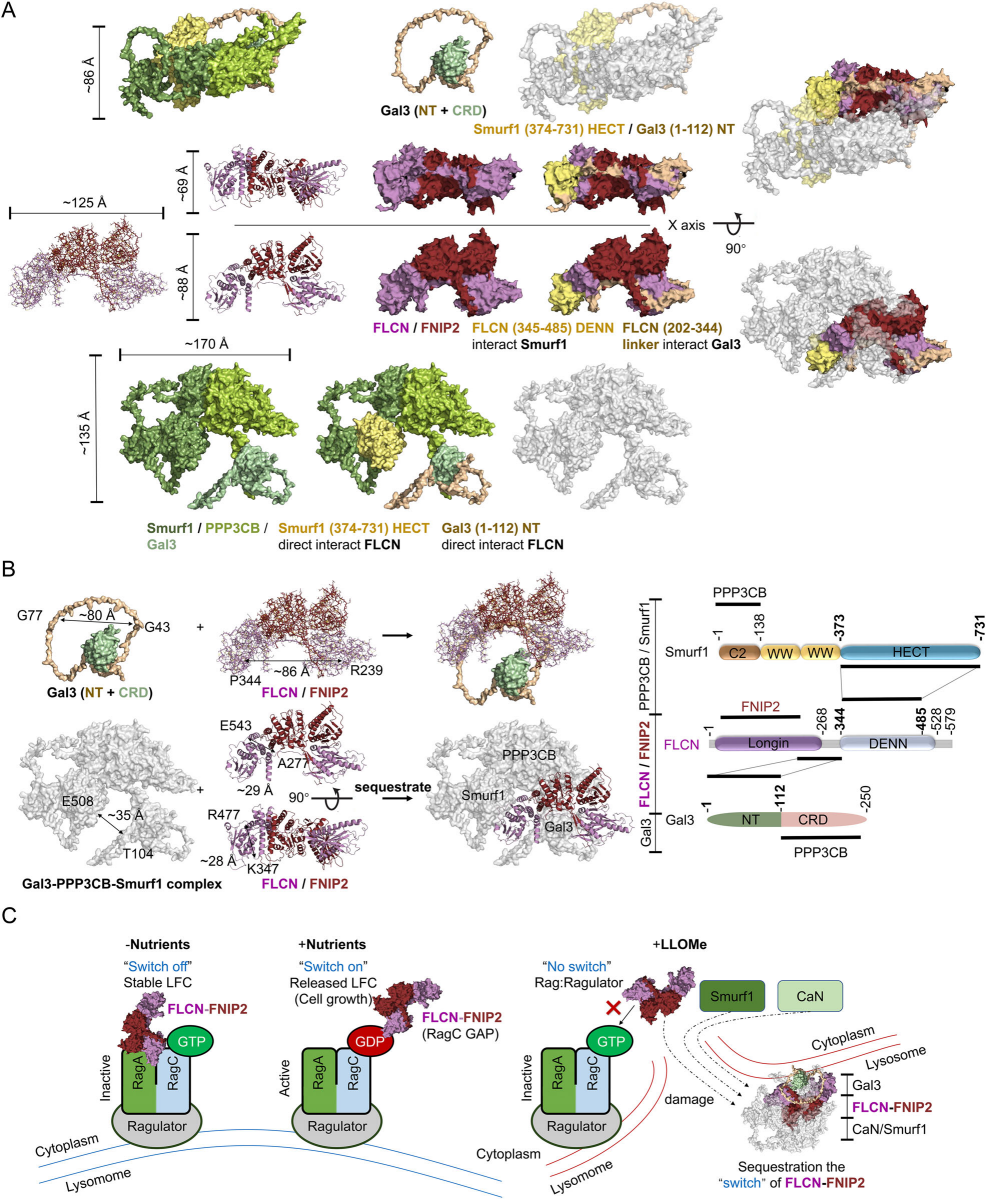
Structure basis for the sequestration of the FLCN‐FNIPs by Gal3‐CaN‐Smurf1 Complex. A) Atomic model, cartoon model and domain assignment for the Gal3‐ PPP3CB‐Smurf1 complex and FLCN‐FNIP2 complex. Subunits of the Gal3‐PPP3CB‐Smurf1 complex and FLCN‐FNIP2 complex are colored as following: Smurf1: smudge; PPP3CB: limon; Gal3: palegreen; FLCN: violet; FNIP2: ruby. B) Structure and domain organization for the Gal3‐PPP3CB‐Smurf1 complex with FLCN‐FNIP2 interaction. The Gal3 NT domain forms a loop around the CRD, with a distance of ≈80 Å between residues G43 and G77. In the FLCN‐FNIP2 complex, distances are ≈86 Å between P344 and R239, ≈29 Å between E543 and A277, and ≈28 Å between R477 and K347. The distance in Gal3‐PPP3CB‐Smurf1 complex between E508 and T104 is ≈35 Å. On the right panel, Inter‐subunit interactions are shown by black lines between domains. Smurf1 is shown with key domains color‐coded: the C2 domain (brown, residues 1–138), two WW domains (yellow, residues 138–373), and the HECT domain (blue, residues 374–731). The FLCN is depicted with two major domains: the Longin domain (purple, residues 1–268) and the DENN domain (light blue, residues 344–485). The linker region between residues 268–344 (not colored) mediates interactions with Smurf1 and Gal3. Gal3 is represented with its NT (green, residues 1–112) and CRD (pink, residues 112–250). C) Structure‐based model for the regulation of the FLCN‐FNIP2 complex by nutrient availability and lysosomal damage. Under nutrient‐deprived conditions, the Rag GTPase complex is in its inactive form (RagA‐GDP/RagC‐GTP), leading to the “switch off” state where the FLCN‐FNIP2 complex is stable and associated with the Ragulator. In nutrient‐rich conditions, the Rag GTPase switches to an active state (RagA‐GTP/RagC‐GDP), which triggers the “switch on” state, releasing the FLCN‐FNIP2 complex and promoting cell growth. Upon exposure to LLOMe, the FLCN‐FNIP2 complex is sequestered by the Gal3‐CaN‐Smurf1 complex.

We utilized Alphafold2^[^ ^24^ ^]^ to simulate the 3D structure of the Gal3‐CaN‐Smurf1 complex, revealing an elongated shape of 170 Å in the longest dimension. The structure features a prominent groove between Gal3 and the PPP3CB‐Smurf1 subcomplex (**Figure** **7**A). To investigate potential interaction structure, we compared this model to the FLCN‐FNIP2 complex structure obtained via cryo‐electron microscopy at a resolution of 3.6 Å (PDB: 6ULG^[^ ^25^ ^]^). The Gal3 N‐terminal domain, ≈80 Å in length, fits within the annular diameter of the FLCN‐FNIP2 complex, which exceeds 86 Å (Figure 7B). Notably, the groove within the Gal3‐CaN‐Smurf1 complex, measuring ≈35 Å, is sufficient to accommodate the FLCN‐FNIP2 complex (Figure 7B). Altogether, FLCN‐FNIP2 acts as a regulatory switch for Rag‐Ragulator activity by forming either stable LFC (inactive form) and a released LFC (active form) under the physiological starvation condition. Here, we hypothesis the Gal3‐CaN‐Smurf1 complex sequesters the “switch” in response to lysosomal membrane damage (Figure 7C).

### 2.8 FLCN‐K462R and FNIP2‐K466R Mutations Reduce its Sequestration Affinity with the Gal3‐CaN‐Smurf1 Complex

**Figure 8:**
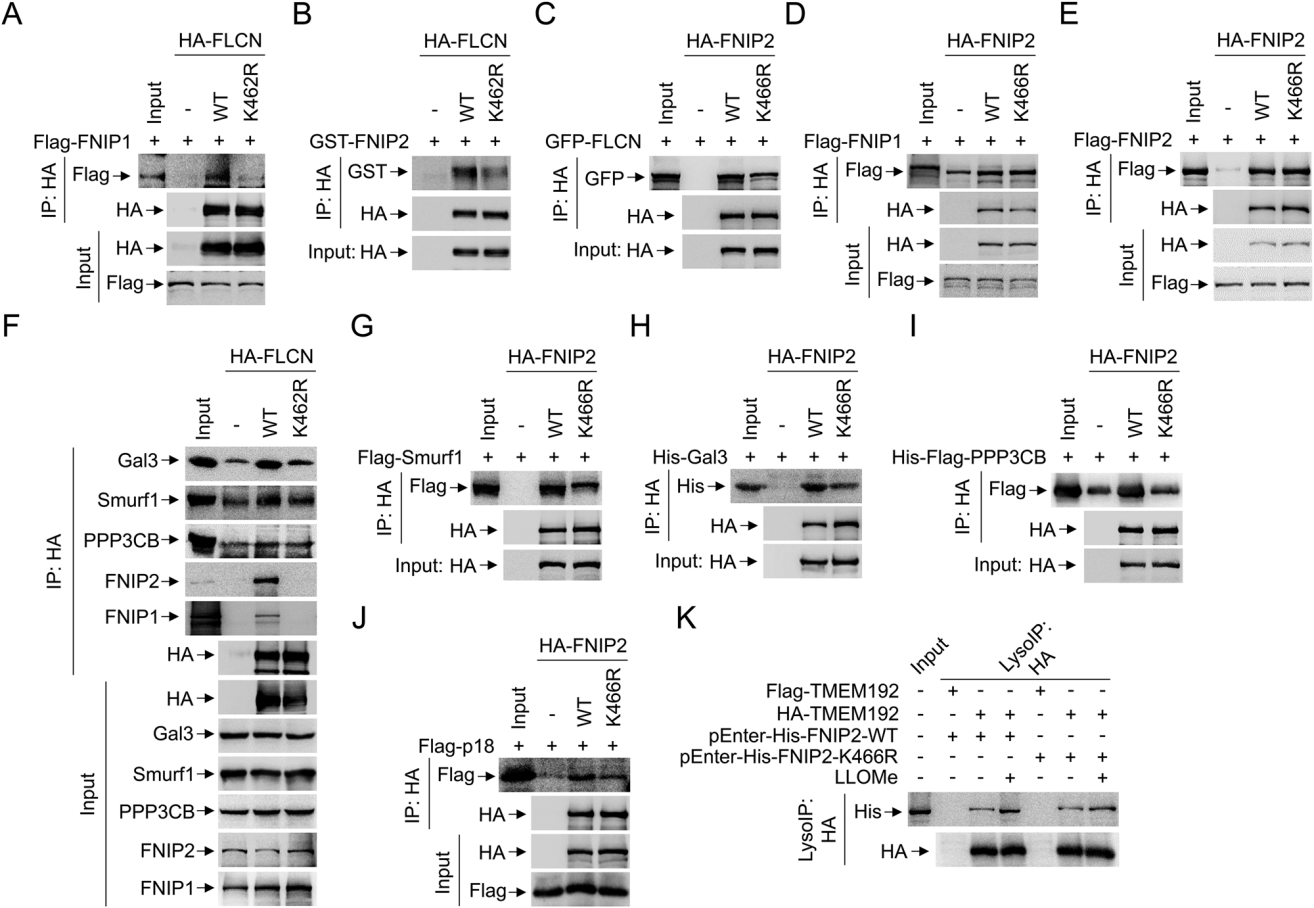
Mutations in FLCN‐FNIP2 (FLCN‐K462R and FNIP2‐K466R) reduce its binding affinity with the Gal3‐CaN‐Smurf1 complex. A) Co‐IP analysis of the interaction between HA‐FLCN constructs and Flag‐FNIP1 expressed in HEK293 cells. B) Co‐IP analysis of the interaction between HA‐FLCN constructs expressed in HEK293 cells and GST‐FNIP2 purified from E. coli. C–E) Co‐IP analysis of the interaction between HA‐FNIP2 constructs and GFP‐FLCN (C), Flag‐FNIP1 (D), or Flag‐FNIP2 (E) expressed in HEK293 cells. F) Co‐IP analysis of the interaction between HA‐FLCN constructs and indicated endogenous proteins expressed in HEK293 cells. G) Co‐IP analysis of the interaction between HA‐FNIP2 constructs and Flag‐Smurf1 expressed in HEK293 cells. H,I) Co‐IP analysis of the interaction between HA‐FNIP2 constructs expressed in HEK293 cells and His‐Gal3 (H) or His‐Flag‐PPP3CB (I) purified from E. coli. J) Co‐IP analysis of the interaction between HA‐FNIP2 constructs and Flag‐p18 expressed in HEK293 cells. K) HEK293 cells were transfected with pEnter‐His‐FNIP2‐WT or pEnter‐His‐FNIP2‐K466R and HA‐TMEM192 or Flag‐TMEM192 for 22 h and treated with LLOMe (1 mM, 2 h) or equal volume DMSO. Cell lysates were then LysoIP with HA for immunoblotting with antibodies against HA and His. Data are representative of three independent experiments with three biological replicates.

To investigate the impact of FLCN‐K462R and FNIP2‐K466R mutations on their binding affinity with the Gal3‐CaN‐Smurf1 complex, respectively we performed immunoprecipitation assays. To begin with, we examined whether the FLCN‐ K462R or FNIP2‐K466R mutations affect the stability of the FLCN‐FNIPs complex. The FLCN‐K462R mutation significantly disrupted its interaction with both FNIP1 (**Figure** **8**A) and FNIP2 (Figure 8B). Conversely, the FNIP2‐K466R mutation significantly impeded its interaction with FLCN (Figure 8C), but not with FNIP1 (Figure 8D) or FNIP2 (Figure 8E), indicating that Smurf1‐mediated ubiquitylation of FLCN and FNIP2 contributes to FLCN‐FNIPs stability. Furthermore, both the FLCN K462R (Figure 8F) and FNIP2‐K466R (Figure 8G–I) mutations significantly reduced their interactions with the Gal3‐CaN‐Smurf1 complex. Similarly, the FNIP2‐K466R mutation markedly decreased its interaction with Rag‐Ragulator (Figure 8J). Notably, the FNIP2‐K466R mutation significantly impaired its lysosomal localization in response to LLOMe, as evidenced by that FNIP2‐K466R, but not FNIP2‐WT, inhibits its interaction with the lysosomal HA‐TMEM192 (Figure 8K).

### 2.9 LLOMe Enhances the Interaction between the Gal3‐CaN‐Smurf1 Complex and FLCN‐FNIPs

**Figure 9:**
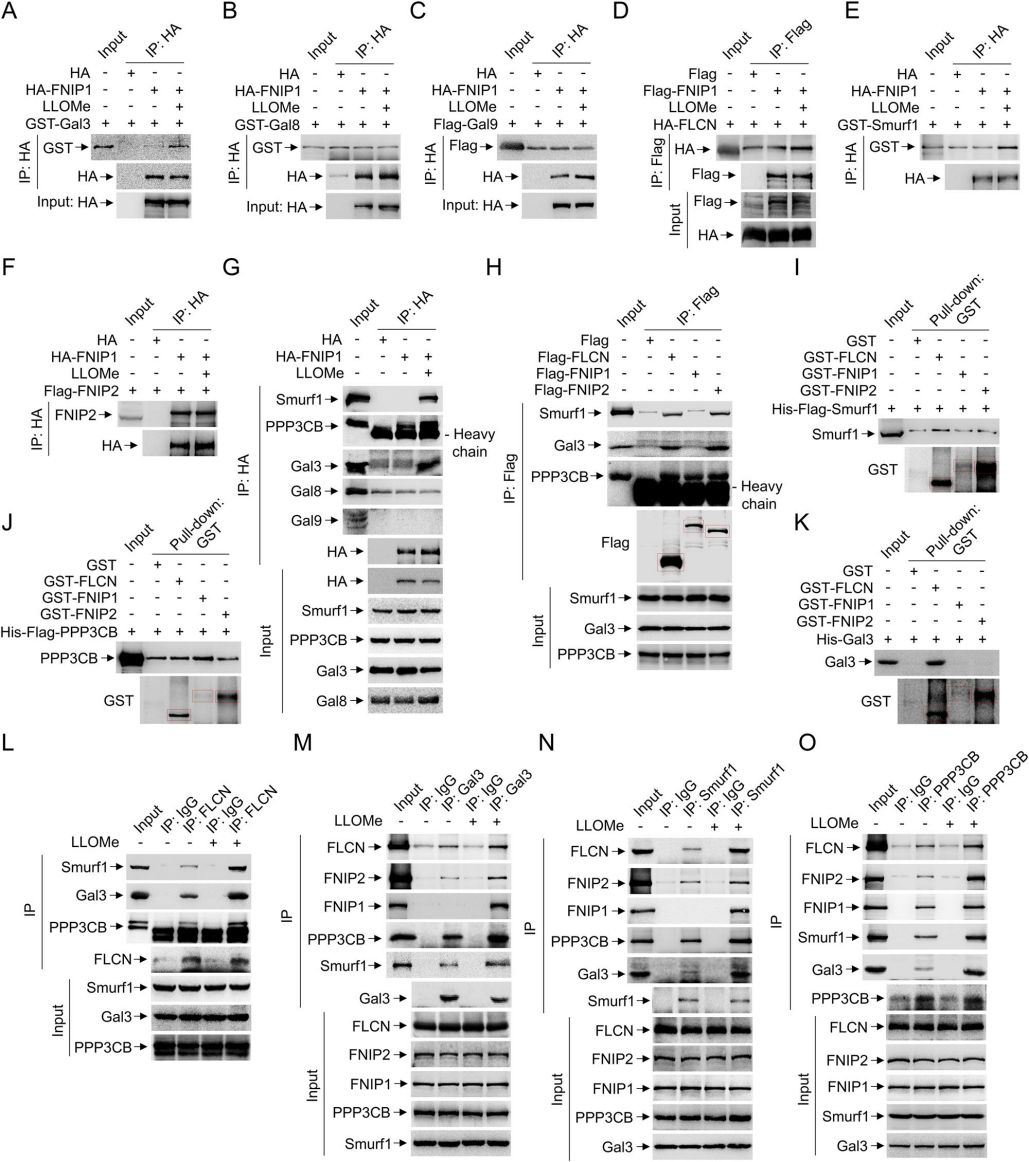
LLOMe enhances the interaction between the Gal3‐CaN‐Smurf1 complex and FLCN‐FNIPs. A,B) Co‐IP analysis of the interaction between HA‐FNIP1 expressed in HEK293 cells and GST‐Gal3 (A) or GST‐Gal8 (B) purified from E. coli. Cells were treated with LLOMe (1 mM, 2 h) or equal volume DMSO. C) Co‐IP analysis of the interaction between HA‐FNIP1 and Flag‐Gal9 expressed in HEK293cells. Cells were treated with LLOMe (1mM, 2h) or equal volume DMSO. D) Co‐IP analysis of the interaction between Flag‐FNIP1 and HA‐FLCN expressed in HEK293 cells. E) Co‐IP analysis of the interaction between HA‐FNIP1 expressed in HEK293 cells and GST‐Smurf1 purified from E. coli. F,G) Co‐IP analysis of the interaction between HA‐FNIP1 and Flag‐FNIP2 (F) or indicated endogenous proteins (G) expressed in HEK293 cells treated with or without LLOMe (1 mM, 2 h). H) Co‐IP analysis of the interaction between Flag‐FLCN, Flag‐FNIP1 or Flag‐FNIP2 and indicated endogenous proteins expressed in HEK293 cells. I–K) GST pulldown analysis of the interaction between GST‐FLCN, GST‐FNIP1 or GST‐FNIP2 and His‐Flag‐Smurf1 (I), His‐Flag‐PPP3CB (J) or His‐Gal3 (K) purified from E. coli. L–O) Co‐IP analysis of the interaction between endogenous FLCN (L), Gal3 (M), Smurf1 (N), or PPP3CB (O) and indicated endogenous proteins in HEK293 cells. Data are representative of three independent experiments with three biological replicates.

Next, to analyze the impact of lysosomal damage on the interaction between the Gal3‐CaN‐Smurf1 complex and FLCN‐FNIPs, we conducted a series of interaction assays. Initially, we observed that Gal3 (**Figure** **9**A), but not Gal8 (Figure 9B) or Gal9 (Figure 9C), significantly interacts with FNIP1 in response to LLOMe. Similarly, LLOMe treatment enhanced the interaction of FNIP1 with FLCN (Figure 9D) and Smurf1 (Figure 9E), but not with FNIP2 (Figure 9F). Additionally, FNIP1 showed a marked interaction with the Gal3‐CaN‐Smurf1 complex upon LLOMe treatment (Figure 9G).

To dissect the individual contributions of FLCN‐FNIPs to the interaction with the Gal3‐CaN‐Smurf1 complex under basal conditions, we performed immunoprecipitation assays using individual components of the FLCN‐FNIPs complex. Interestingly, we found that both FLCN and FNIPs interacted with PPP3CB individually. Additionally, Flag‐FLCN and Flag‐FNIP2, but not Flag‐FNIP1, interacted with Smurf1 and Gal3 in *ex vivo* assays (Figure 9H). Next, using purified Smurf1, PPP3CB, and Gal3 proteins, we examined their direct interactions with individual FLCN‐FNIPs components. We identified that: 1) His‐Flag‐Smurf1 directly interacts with GST‐FLCN (Figure 9I); 2) His‐Flag‐PPP3CB directly interacts with GST‐FNIP1 (Figure 9J); 3) His‐Flag‐Gal3 directly interacts with GST‐FLCN (Figure 9K). Additionally, immunoprecipitation showed significant enhanced its binding affinity within the Gal3‐CaN‐Smurf1 complex in response to LLOMe (Figure 9L). Conversely, immunoprecipitated Gal3‐CaN‐Smurf1 complex significantly enhanced its binding affinity with FLCN‐FNIPs in response to LLOMe (Figure 9M–O). Altogether, these findings suggest that LLOMe promotes the stability and interaction affinity of both the FLCN‐FNIPs and Gal3‐CaN‐Smurf1 complexes.

To verify the role of the Gal3‐CaN‐Smurf1 complex in the sequestration of FLCN‐FNIPs, we either overexpressed or knocked down components of the Gal3‐CaN‐Smurf1 complex. Consistently, overexpression of any components of the Gal3‐CaN‐Smurf1 complex promoted the sequestration of FLCN (Figure S5A–C, Supporting Information). Conversely, knocking down Gal3, Smurf1, or PPP3CB significantly reduced the sequestration of FLCN (Figure S5D–F, Supporting Information). To identify which component of the Gal3‐CaN‐Smurf1 complex contributes most to the stability of the FLCN‐FNIPs complex, we overexpressed FLCN along with FNIP2. Interestingly, Smurf1 was found to be the major contributor to the formation of the FLCN‐FNIP2 complex. This was evidenced by the fact that the overexpression of GFP‐Smurf1, but not GFP‐PPP3CB or GFP‐Gal3, which markedly enhanced the interaction between FLCN and FNIP2 (Figure S5G, Supporting Information). Conversely, knocking down Smurf1, but not the PPP3CB or Gal3, significantly reduced the interaction between FLCN and FNIP2 (Figure S5H, Supporting Information). These data further indicate that Smurf1‐mediated ubiquitylation of FLCN and FNIP2 plays a critical role in the FLCN‐FNIP2 interaction.

### 2.10 Smurf1 Interacts with and Ubiquitylates TFEB

**Figure 10:**
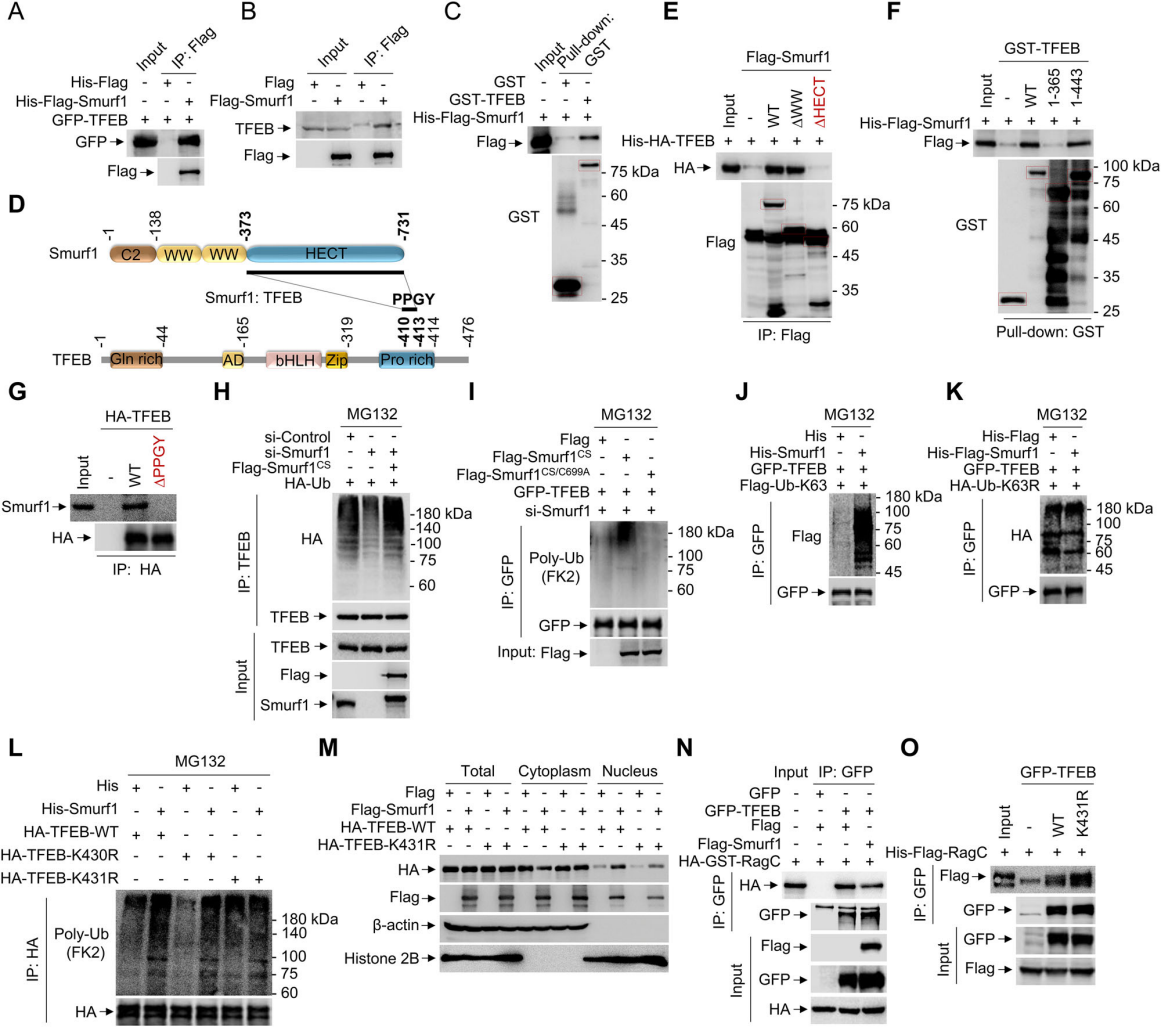
Smurf1 interacts with and ubiquitylates TFEB. A) Co‐IP analysis of the interaction between GFP‐TFEB expressed in HEK293 cells and His‐Flag‐Smurf1 purified from E. coli. B) Co‐IP analysis of the interaction between Flag‐Smurf1 and endogenous TFEB expressed in HEK293 cells. C) GST Pull‐down analysis of the interaction between GST‐TFEB and His‐Flag‐Smurf1 purified from E. coli. D) Schematic diagram of mapping the direct interaction between Smurf1 and TFEB. E) Co‐IP analysis of the interaction between Flag‐Smurf1 constructs expressed in HEK293 cells and His‐HA‐TFEB purified from E. coli. F) Co‐IP analysis of the interaction between GST‐TFEB constructs and His‐Flag‐Smurf1 purified from E. coli. G) Co‐IP analysis of the interaction between HA‐TFEB constructs and endogenous Smurf1 expressed in HEK293 cells. H,I) HEK293 cells were transfected with indicated plasmids and siRNAs and then treated with MG132 (10 µM) for another 12 h. Cell lysates were subjected to IP with TFEB (H) or GFP (I) for immunoblotting with indicated antibodies. J) HEK293 cells were transfected with GFP‐TFEB and Flag‐Ub‐K63 and then treated with MG132 (10 µM) for another 12 h. Cell lysates were then incubated with or without His‐Smurf1 and IP with GFP for immunoblotting with antibodies against Flag and GFP. K) HEK293 cells were transfected with GFP‐TFEB and HA‐Ub‐K63R and then treated with MG132 (10 µM) for another 12 h. Cell lysates were then incubated with or without His‐Flag‐Smurf1 and IP with GFP for immunoblotting with antibodies against HA and GFP. L) HEK293 cells were transfected with HA‐TFEB constructs and then treated with MG132 (10 µM) for another 12 h. Cell lysates were then incubated with or without His‐Smurf1 and IP with HA for immunoblotting with antibodies against Ub and HA. M) HEK293 cells were transfected with HA‐TFEB constructs and Flag‐Smurf1 for 24 h and then subjected to nuclear and cytoplasmic separation assay and western blotting analysis using anti‐HA, anti‐Flag, anti‐β‐actin and anti‐Histone 2B antibodies. N) Co‐IP analysis of the interaction between GFP‐TFEB and HA‐GST‐RagC expressed in HEK293 cells. O) Co‐IP analysis of the interaction between GFP‐TFEB constructed expressed in HEK293 cells and His‐Flag‐RagC purified from E. coli. Data are representative of three independent experiments with three biological replicates.

Surprisingly, we found that Smurf1 also directly interacts with TFEB in LLOMe or Smurf1 E3 ligase activity independent manner (**Figure** **10**A–C and Figure 6A,B). TFEB comprises a glutamine‐rich (Gln‐rich) domain, a basic helix‐loop‐helix (bHLH) domain, a leucine zipper (Zip) domain, and a proline‐rich (Pro‐rich) domain. We identified that the HECT domain of Smurf1 interacts with the Pro‐rich domain (residues 366–443) of TFEB (Figure 10D–F; Figure S6C,D, Supporting Information). Specifically, Smurf1 typically recognizes the PPxY motif, which TFEB contains a PPGY (residues 410–413) motif. To test whether their interaction depends on the PPGY motif, we generated a truncated mutation of TFEB. The results showed that the PPGY motif is necessary for Smurf1 binding, as no association was detected between Smurf1 and TFEB‐ΔPPGY (Figure 10G). Additionally, overexpression of Smurf1 significantly promotes, as well as suppression of Smurf1 blocks, the ubiquitination of TFEB compared to the control under MG132 treatment (Figure 10H; Figure S6E, Supporting Information). Overexpression of Smurf1, but not the catalytically inactive Smurf1‐C699A mutant, rescued the ubiquitination of TFEB in Smurf1‐deleted cells (Figure 10H,I). To determine which type of lysine ubiquitin chain is added to TFEB by Smurf1, we performed immunoprecipitation assays in the presence of different ubiquitin variants. Interestingly, our data showed that Smurf1 increased K63‐linked (Figure 10J,K), but not K48‐linked (Figure S6F,G, Supporting Information), ubiquitination of TFEB.

The HECT domain of Smurf1 ubiquitinates TFEB both in vitro and *ex vivo* (Figure S6H,I, Supporting Information). Additionally, the potential ubiquitination sites of TFEB mediated by Smurf1 is located within residues 366–443, as evidenced by the significant decrease in ubiquitination levels in a TFEB construct deleted with residues 1–365 (Figure S6J, Supporting Information). We then generated point mutants K430R and K431R within the 366–443 amino acid region and found that K431R, but not K430R, failed to be ubiquitinated by Smurf1 (Figure 10L). Furthermore, the K431R mutation resulted in decreased TFEB nuclear import (Figure 10M), suggesting that Smurf1‐mediated ubiquitination of TFEB at K431 is critical for TFEB nuclear translocation. These results demonstrate that Smurf1 ubiquitinates TFEB at K431. Importantly, Smurf1 overexpression impaired the binding affinity between TFEB and RagC (Figure 10N), and the TFEB K431R mutant significantly enhanced the TFEB‐RagC interaction (Figure 10O). These data collectively imply that Smurf1‐mediated ubiquitination of TFEB at K431 contributes to the dissociation of TFEB from RagC.

### 2.11 FLCN‐FNIPs Stabilizes Gal3‐PPP3CB‐Smurf1 Complex

**Figure 11:**
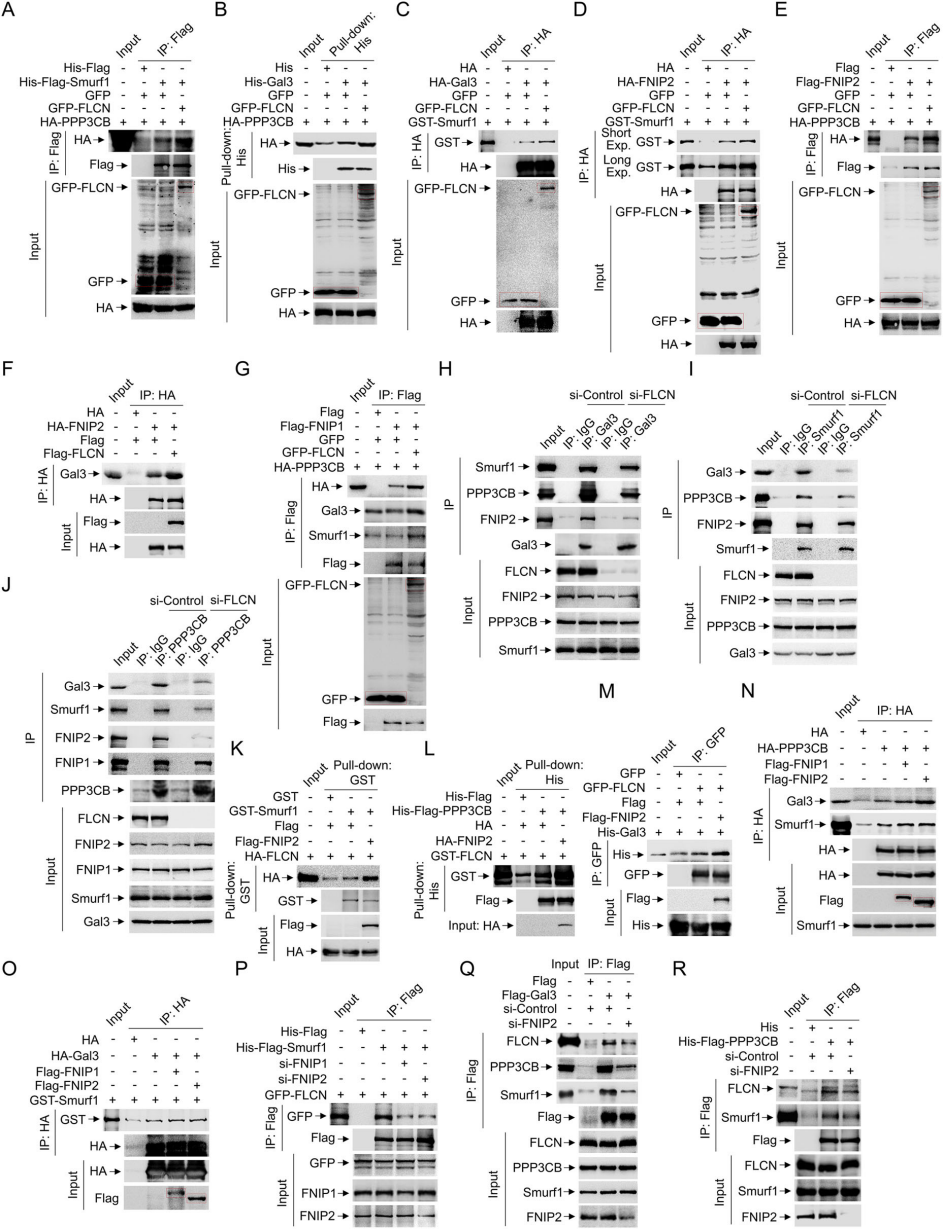
FLCN‐FNIPs stabilizes Gal3‐PPP3CB‐Smurf1 complex. A) Co‐IP analysis of the interaction between HA‐PPP3CB expressed in HEK293 cells and His‐Flag‐Smurf1 purified from E. coli. B) His Pull‐down analysis of the interaction between His‐Gal3 purified from E. coli and HA‐PPP3CB expressed in HEK293 cells. C) Co‐IP analysis of the interaction between HA‐Gal3 expressed in HEK293 cells and GST‐Smurf1 purified from E. coli with or without FLCN. D) Co‐IP analysis of the interaction between HA‐FNIP2 expressed in HEK293 cells and GST‐Smurf1 purified from E. coli withe or without FLCN. E) Co‐IP analysis of the interaction between Flag‐FNIP2 and HA‐PPP3CB expressed in HEK293 cells. F) Co‐IP analysis of the interaction between HA‐FNIP2 and endogenous Gal3 expressed in HEK293 cells. G) Co‐IP analysis of the interaction between Flag‐FNIP1 and HA‐PPP3CB expressed in HEK293 cells. H–J) Co‐IP analysis of the interaction between endogenous Gal3 (H), Smurf1 (I), or PPP3CB (J) and indicated endogenous proteins in HEK293 cells. K) GST Pull‐down analysis of the interaction between GST‐Smurf1 purified from E. coli and HA‐FLCN expressed in HEK293 cells. L) His Pull‐down analysis of the interaction between His‐Flag‐PPP3CB and GST‐FLCN purified from E. coli. M) Co‐IP analysis of the interaction between GFP‐FLCN expressed in HEK293 cells and His‐Gal3 purified from E. coli. N) Co‐IP analysis of the interaction between HA‐PPP3CB and indicated endogenous proteins expressed in HEK293 cells. O) Co‐IP analysis of the interaction between HA‐Gal3 expressed in HEK293 cells and GST‐Smurf1 purified from E. coli. P) Co‐IP analysis of the interaction between GFP‐FLCN expressed in HEK293 cells and His‐Flag‐Smurf1 purified from E. coli. Q) Co‐IP analysis of the interaction between Flag‐Gal3 and indicated endogenous proteins expressed in HEK293 cells. R) Co‐IP analysis of the interaction between His‐Flag‐PPP3CB purified from E. coli and indicated endogenous proteins expressed in HEK293 cells. Data are representative of three independent experiments with three biological replicates.

We next investigated the role of FLCN‐FNIPs in stabilizing the Gal3‐CaN‐Smurf1 complex. Indeed, FLCN promotes the binding affinity within the Gal3‐CaN‐Smurf1 complex, evidenced by the fact that overexpression of GFP‐FLCN significantly enhanced the interaction between any two components of the Gal3‐CaN‐Smurf1 complex (**Figure** **11**A–C). Furthermore, FLCN overexpression promoted the interaction of FNIP2 with GST‐Smurf1 (Figure 11D), HA‐PPP3CB (Figure 11E), and Gal3 (Figure 11F). Similarly, FLCN overexpression enhanced the interaction of FNIP1 with HA‐PPP3CB (Figure 11G). Conversely, knocking down FLCN significantly decreased the interaction between any two components of the Gal3‐CaN‐Smurf1 complex (Figure 11H–J).

Additionally, overexpression of FNIP2 significantly enhanced the interaction of FLCN with each component of the Gal3‐CaN‐Smurf1 complex (Figure 11K–M). Overexpression of Flag‐FNIP1 or Flag‐FNIP2 promoted the interaction between HA‐PPP3CB and endogenous Gal3 or Smurf1 (Figure 11N), or GST‐Smurf1 (Figure 11O). Conversely, knocking down FNIP1 or FNIP2 decreased the interaction of Smurf1 with FLCN (Figure 11P), Gal3 with FLCN, PPP3CB, and Smurf1 (Figure 11Q), as well as the interaction of PPP3CB with FLCN and Smurf1 (Figure 11R). Collectively, these data strongly support the notion that FLCN‐FNIPs facilitate the stability of the Gal3‐PPP3CB‐Smurf1 complex.

## 3 Summary

### 3.1 Gal3‐CaN‐Smurf1 Complex Sequestrates FLCN‐FNIPs to Facilitate TFEB Activation in Response to Endomembrane Damage

**Figure 12:**
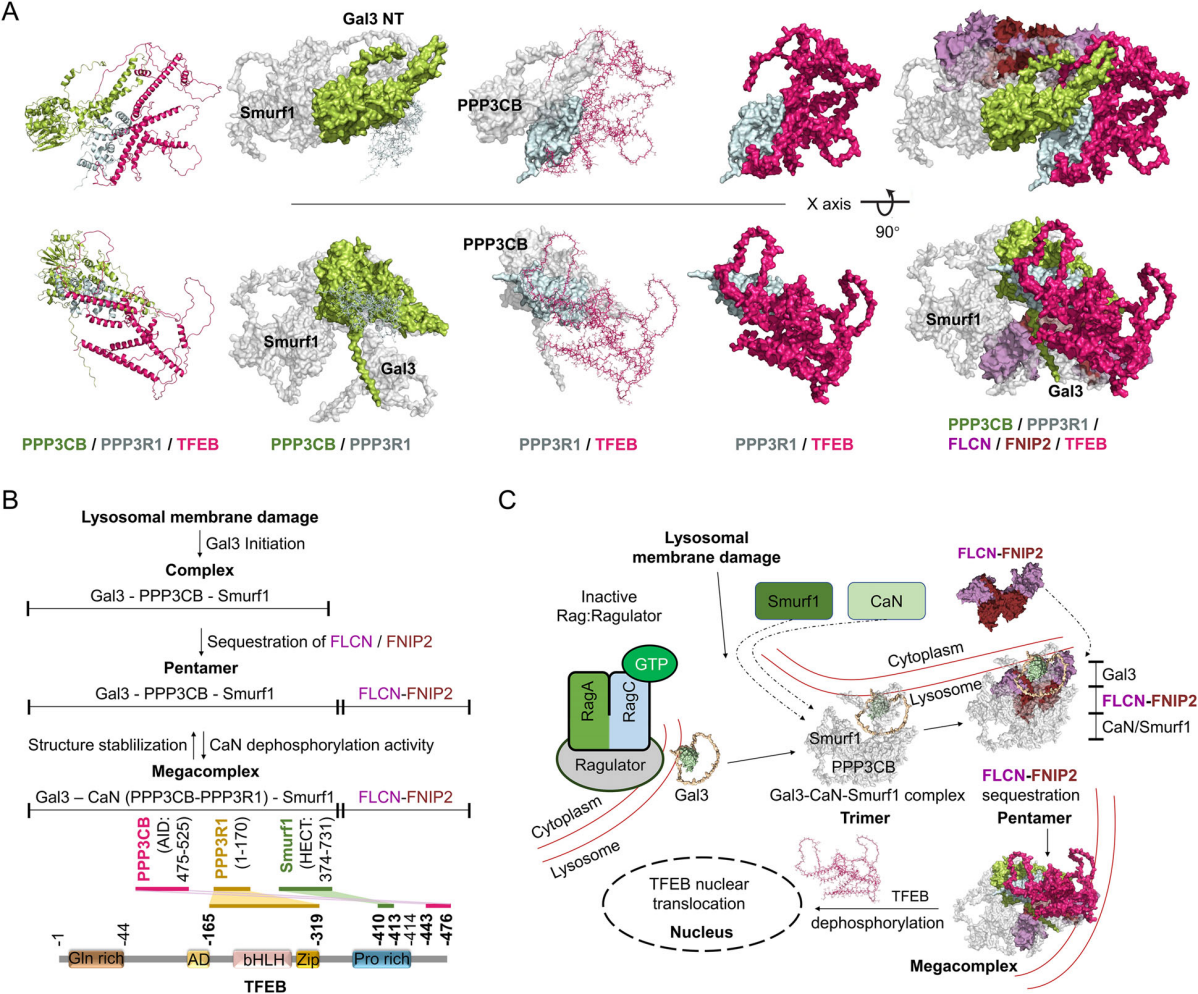
Gal3‐CaN‐Smurf1 complex sequestrates FLCN‐FNIPs to facilitate TFEB activation in response to endomembrane damage. A) Atomic model, cartoon model and domain assignment for the PPP3CB‐PPP3R1‐TFEB complex. Subunits of the complex are colored as following: PPP3CB: limon; PPP3R1: palecyan; TFEB: hotpink. B) Schematic of lysosomal membrane damage and subsequent steps in complex formation. Gal3 initiation the formation of the complex and the core complex consists of Gal3, PPP3CB, and Smurf1, which leading to the sequestration of the FLCN‐FNIP2 complex. Further interaction results in the formation of a pentamer complex, stabilizing the sequestration of FLCN‐FNIP2, critical for its regulation. The structure stabilizes with additional components including CaN (PPP3CB‐PPP3R1) formatting the megacomplex. At the bottom, TFEB is depicted with key structure domains, including Gln rich, AD, Bhlh, Zip, and Pro rich regions. Inter‐subunit interactions are shown by colored bars between domains. (C) Structure‐based model for the lysosomal membrane damage response and the assembly of the Gal3‐PPP3CB‐Smurf1 complex.

We utilized Alphafold2^[^ ^24^ ^]^ to simulate the 3D structure of the CaN (PPP3CB/PPP3R1)‐TFEB complex. The structure features a prominent rivet of PPP3R1 between PPP3CB and TFEB (**Figure** **12**A). Importantly, we identified the FLCN‐FNIPs from damaged lysosome induces the stability of Gal3‐CaN‐Smurf1 complex and the recruitment to lysosome during LLOMe treatment. The Gal3‐CaN‐Smurf1 complex interacts with FLCN‐FNIPs, further facilitating the formation of Gal3‐CaN‐Smurf1 and FLCN‐FNIPs megacomplex that is essential for TFEB activation (Figure 12B). Our research demonstrates that endomembrane damage leads to the formation of the Gal3‐CaN‐Smurf1 complex, which in turn sequestrates to FLCN‐FNIPs. This interaction results in the dephosphorylation of TFEB by CaN and the release of TFEB from its cytoplasmic retention, allowing its translocation to the nucleus for subsequent expression of TFEB target genes (Figure 12C).

## 4 Discussion

MTORC1 is the master controller for protein synthesis by interconnected sensing the status of nutrition via directly phosphorylation of the canonical substates S6K and 4E‐BP1. TFEB (S211) can be phosphorylated by mTORC1 kinase at the lysosomal surface and binds to cytosolic chaperone 14‐3‐3, resulting in its sequestration in the cytoplasm.^[^ ^6^, ^26^ ^]^ Previously, germline loss‐of‐function mutations in *FLCN* hyperactivate TFEB in Birt‐Hogg‐Dubé syndrome,^[^ ^2^, ^27^ ^]^ a rare abundant benign dermal hamartoma‐like tumors with high incidence of renal cell carcinoma (RCC).^[^ ^28^ ^]^ The existence of the substrate‐specific mechanism explains that loss of FLCN unleashes TFEB to the nucleus, but has no direct impact on canonical mTORC1 signaling.^[^ ^2^ ^]^ The mTORC1 signaling can generally separate into FLCN‐independent (canonical substates S6K and 4E‐BP1) and FLCN dependent (noncanonical substate TFEB) mechanism, thus explains the apparent paradoxical development of tumors with high mTORC1 activity in BHD patients. Our data provide compelling evidence that Gal3‐CaN‐Smurf1 complex dependent membrane sequestration of the FLCN‐FNIPs complex uncouples its regulation of RagC/D, revealing a new paradigm of TFEB activation distinct from LFC formation during nutrient starvation. Gal3‐CaN‐Smurf1 complex dependent TFEB activation is permissive with mTORC1 activity, offering new insights into strategies to enhance lysosomal biogenesis. The complex orchestrates a multi‐level regulatory mechanism that ensures the temporally precise activation of TFEB through the ubiquitylation of its interacting partners. First, Smurf1‐mediated K63 ubiquitylate both FLCN at K462 and FNIP2 at K466 facilitates the interaction between FLCN‐FNIP2 and Gal3‐CaN‐Smurf1 complex. Second, that Smurf1 interacts with and ubiquitylates TFEB blocks the binding affinity between TFEB and RagC. Third, sequestrated FLCN‐FNIPs feedback stabilizes Gal3‐CaN‐Smurf1 complex.

Gal3 can rapidly sensor and bind to exposed glycans on the luminal side of the lysosome in response to membrane rupture, which initiate the lysophagy process to target damaged lysosomes.^[^ ^11^, ^13^ ^]^ Previously, it is generally believed that Gal3‐CaN‐Smurf1 complex formation localized in facing the lysosomal lumen,^[^ ^4^ ^]^ while Rag‐Ragulator localized to outer leaflet^[^ ^29^ ^]^ on the lysosomal membrane. In this manuscript, to distinguish whether the recruited FLCN‐FNIPs is localized to the inner or outer leaflet on in response to lysosomal damage, we knocked down either p18 or Gal3. We found Gal3, but not p18, is required for the sequestration of FLCN‐FNIPs at the lysosomal membrane in response to LLOMe (Figure 3E,F). We propose the recruitment of the pentamer complex to the lysosomal membrane. However, Further evidence is needed to identify whether the pentamer complex stays in the inner leaflet of the lysosomal membrane for TFEB activation and/or works as stress granules plug to stabilize damaged endolysosomal membranes.^[^ ^17^ ^]^ Lysosomes that undergo severe lysosomal membrane permeabilization (LMP) can be selectively degraded through lysophagy, while mild LMP can be repaired more quickly and directly via the transport of the required endosomal sorting complex (ESCRT). ESCRT‐mediated repair function soon after lysosomal damage seals the damaged lysosome.^[^ ^30^ ^]^ It has also been reported ESCRT‐mediated repair precede lysophagy.^[^ ^31^ ^]^ However, in the absence of ESCRT subunits, cells are still able to repair lysosomal damage, indicating that there are other repair mechanisms present in lysosomes. Further study identifies the core PITT pathway mechanism by which cells rapidly repair lysosomal damage – lysosomal damage triggers a specific lipid signal, PI4P, on the lysosomal surface. This signal promotes strong interactions between the endoplasmic reticulum and lysosomes, subsequently activates the transport of multiple lipids from the endoplasmic reticulum to the lysosome.^[^ ^32^ ^]^ We hypothesis Smurf1 mediates selectively lysophagy in response to severe lysosomal membrane permeabilization (LMP). Specifically, calcium fluxes are generally believed to occur in response to lysosomal membrane permeabilization (LMP). Upon failure of the repair mechanisms, lysophagy is activated. In such instances, lysosomal hydrolases may re‐equilibrate during severe lysosomal membrane damage. These pentameric complexes freely migrate within the lumen, facilitating selective autophagy.

The structural insights into the FLCN‐FNIPs/Gal3‐CaN‐Smurf1 interface broadly serve to coordinate lysosomal interconnected stages how cell switch from cell growth status to autophagic degradation of damaged lysosome in cope with endolysosomal stress. Our structure model of TFEB activator reveals how Gal3‐CaN‐Smurf1 and FLCN‐FNIPs assemble into megacomplex by means of an intricate domain arrangement, as well as how they interact with the TFEB in their active state. It serves as the structure basis for FLCN‐FNIPs sequestration at the lysosomal membrane (Gal3‐CaN‐Smurf1) under endolysosomal damage and explains our findings from immunoprecipitation and biological assay. First, Rag‐Ragulator/ FLCN‐FNIPs regulates a subset of mTORC1 substrates, including TFEB, by controlling the RagC‐GAP activity of FLCN.^[^ ^1^ ^]^ The ability of FLCN‐FNIPs to interact with RagC, the component of Rag‐Ragulator, in both the inactive stable LFC and transient interaction (released LFC) states, which is critical for TFEB phosphorylation in response to nutrients.^[^ ^1^ ^]^ We identified lysosomal membrane complex Gal3‐CaN‐Smurf1 overwhelmingly compete with Rag‐Ragulator to sequestrate FLCN‐FNIPs for TFEB dephosphorylation in response to lysosomal damage.

The intricate network involving the Gal3‐CaN‐Smurf1 complex and FLCN‐FNIPs emerges as a central regulatory hub controlling TFEB activation in response to lysosomal damage. The specificity of the interactions and the role of ubiquitylation in this context underline the importance of post‐translational modifications in cellular stress responses. Moreover, elevated activity and permeability of lysosomes has been noted in PDACs,^[^ ^33^ ^]^ and our study suggests a mechanism to explain TFEB nuclear localization despite nutrient‐replete and mTOR‐active conditions.^[^ ^23^ ^]^ Indeed, oncogenic signals might take advantage of involvement of FLCN‐FNIPs membrane sequestration in endolysosomal stress setting to drive TFEB‐dependent tumor growth. The “enhanced capabilities” acquired by cells to adapt to the rapid proliferation of tumors is a key factor in understanding malignant transformation and drug resistance. Tumor cells utilize the highly expressed Smurf1 to link the growth signal to mTOR, providing an evidence for continuously coping with external pressures. Specifically, the high expression of Smurf1 in malignant gliomas promotes the hyperactivation of the mTORC1 pathway by ubiquitin‐mediated degradation of PTEN.^[^ ^34^, ^35^ ^]^ The phosphorylation of p62 mediated by mTORC1 competitively binds to Keap1 (Kelch‐like ECH‐associated protein 1), a negative regulator of the antioxidant transcription factor Nrf2.^[^ ^36^, ^37^ ^]^ The ability of cells to rebalance the homeostasis of the intracellular environment by activating lysosomal autophagy in response to stress perturbations is a key driving force for tumor drug resistance. Smurf1 plays a bidirectional regulation of the mTOR‐TFEB axis in the balance between tumor growth and stress‐induced cell homostasis. Our study provides a potential novel therapeutic target for tumor progression and drug resistance.

## 5 Experimental Section

### Cell Culture and Transfection

Human cell lines HEK293 purchased from the American Type Culture Collection (ATCC) were cultured in Dulbecco's Modified Eagle Medium (DMEM) supplemented with 10% fetal bovine serum (FBS) and 1% penicillin/streptomycin. All cells were grown at 37 °C with 5% CO_2_ in a humidified incubator. Transfections of plasmids were performed using Lipofectamine 2000 (Invitrogen) reagent and siRNA (JTSBIO Co., Ltd (Wuhan, China)) using lipofectamine RNAiMax (Invitrogen) according to the manufacturer's instructions.

### Western Blot

Protein samples were boiled with 2× loading buffer for 10 min and then separated by sodium dodecyl sulfate polyacrylamide gel electrophoresis (SDS‐PAGE). The proteins into gels were transferred to the nitrocellulose membranes in transfer buffer. The membranes with proteins attached to the surface were sealed in 5% non‐fat milk for 1 h and incubated with specific primary antibodies at 4 °C overnight. Next day, the membranes were washed with TBST for 5 min 3 times and then incubated with secondary antibodies at room temperature for 1 h. At last, the membranes were washed with TBST for 5 min 3 times and developed by enhanced chemiluminescence (ECL) assay.

### Immunofluorescence

Cells were seeded onto glass slides placed in 12‐well plate and subjected to experiment treatments. The slides were wash with 1× PBS 3 times and fixed with 4% paraformaldehyde for 15 min and washed 3 times again. Cells attached to the slides were permeated with 0.2% Triton X‐100 for 5 min at room temperature and washed with PBS 3 times. The slides were sealed in 1% BSA for 1 h at room temperature and incubated with specific primary antibodies at 4 °C overnight. Next day, the slides were washed with PBST for 5 min 3 times and then incubated with fluorescent secondary antibodies at room temperature for 1 h at dark. The slides were washed with PBST 3 times again and stained with DAPI for 10 min at dark and mounted with mounting medium. Used confocal microscopy (Nikon A1R(N‐SIM)) to observe the slides and collected images.

### Nuclear and Cytoplasmic Separation

Cells were collected and lysed with 120 µL buffer A (10 mM HEPES [pH 7.9], 10 mM KCl, 1 mM EDTA, 1 mM EGTA, 1 mM DTT) supplemented with 0.5% NP40 for 30 min on ice. Gently blew and mixed the lysate during this period. Took 60 µL of lysate as the total protein sample, and centrifuge the rest portion at 600× g for 15 min at 4 °C. The supernatant removed to a new centrifuge tube, which was the cytoplasmic extract, and the precipitate was washed with buffer A 3–5 times and lysed with 60 µL RIPA buffer, which was the nuclear extract, along with the total protein sample were ultra sonic‐lysed and boiled supplemented with loading buffer. All samples were then detected by western blotting.

### Pull‐Down

The bacterial‐expressed purified proteins were incubated with beads; the pull‐down mixtures were rotated at 4 °C for overnight. The beads were washed with ice‐cold PBS at 3000× g for 2 min 6 times at 4 °C, and then incubated with the second protein at the same conditions. As the first time, the beads were washed with ice‐cold PBS at 3000× g for 2 min 6 times at 4 °C. Finally, the proteins were eluted with 60 µL 2× loading buffer at 98 °C for 10 min and analyzed by western blotting.

### Immunoprecipitation

For immunoprecipitation, cells expressing the indicated tagged protein were rinsed once with cold PBS and lysed in 400 µL immunoprecipitation lysis buffer (50 mM Tris‐HCl, [pH8.0]; 150 mM NaCl; 1% NP40; 0.5% sodium deoxycholate), and lysed cells were sonicated. The supernatants were collected as whole cell lysates after centrifugation at 13 000× g for 15 min. 40 µL of whole cell lysates were saved as input control and the remaining lysates (or bacterial‐expressed negative control and targeted proteins) were incubated with beads. The immunoprecipitation mixtures were rotated at 4 °C for overnight. Then the beads were washed with ice‐cold PBS at 3000× g for 2 min for 6 times at 4 °C, and incubated with the bacterial‐expressed proteins (or indicated native cell lysates) at the same conditions. The beads were washed with ice‐cold PBS at 3000× g for 2 min 6 times at 4 °C and proteins were eluted by 60 µL 2× loading buffer at 98 °C for 10 min. Finally, immunoprecipitation was analyzed by western blotting.

### Molecular Modeling and Docking

FLCN‐FNIP2 and Smurf1‐PPP3CB‐Gal3: The structure of the FLCN‐FNIP2 complex was acquired from the Protein Data Bank (PDB: 6ULG), following research conducted by Shen et al.^[^ ^25^ ^]^ To build the atomic model for the Smurf1‐PPP3CB‐Gal3 complex, the raw amino acid sequences of Smurf1, PPP3CB, and Gal3 to Alphafold2^[^ ^24^ ^]^ initially to generate the 3D model structure of the proteins was submitted. After generation, the Smurf1‐PPP3CB‐Gal3 complex was docked by submitting to the HADDOCK server version 2.4 which is a docking method driven by experimental knowledge.^[^ ^38^, ^39^ ^]^ The docking process for the Smurf1‐PPP3CB‐Gal3 complex into two phases was divided: 1) Initially, the active residues of PPP3CB‐Gal3 were designated based on the experimental data, and the resulting complex was refined to a unified protein structure through script‐based modifications to the original PDB file; 2) Subsequently, docking of the PPP3CB‐Gal3 complex with Smurf1 was performed based on experimentally determined binding sites/regions to set the active residues, ultimately resulting in the creation of the structure of the Smurf1‐PPP3CB‐Gal3 complex. Following the docking procedure, the complex structure was refined and adjusted using the molecular graphics system PyMOL.^[^ ^40^ ^]^

TFEB‐PPP3R1‐PPP3CB‐Smurf1: To build the atomic model for the TFEB‐PPP3R1‐PPP3CB‐Smurf1 complex, the raw amino acid sequences of individual molecular to Alphafold2^[^ ^24^ ^]^ to generate the 3D model structure of the proteins was submitted. The active residue sites based on the experimental determination of direct binding sites between proteins, similar to the docking of the Smurf1‐PPP3CB‐Gal3 complex was identified. The docking of the TFEB‐PPP3R1‐PPP3CB‐Smurf1 complex was divided into three steps: 1) Docking of TFEB with PPP3R1; 2) Docking of the TFEB‐PPP3R1 complex with PPP3CB; 3) Docking of the TFEB‐PPP3R1‐PPP3CB complex with Smurf1. The TFEB‐PPP3R1‐PPP3CB‐Smurf1 complex was docked by the HADDOCK server version 2.4^[^ ^38^, ^39^ ^]^ and visualized with PyMOL.^[^ ^40^ ^]^

### LysoIP

Cells in two 10 cm plates with 90% transfection efficiency were used for each LysoIP. The cells were transfected with HA‐tagged TMEM192 and quickly rinsed twice with pre‐cooled PBS and then resuspended with 1 mL of KPBS (136 mM KCl, 10 mM KH_2_PO_4_, pH 7.25 was adjusted with KOH). The cell suspension was centrifuged at 1000× g for 2 min at 4 °C, and the supernatant was discarded. The precipitated cells were resuspended in 950 µL KPBS, of which 25 µL was retained for whole‐cell lysate processing. The remaining 925 µL suspension was gently homogenized 20 times with a 2 mL homogenizer. The homogenate was then centrifuged at 1000× g for 2 min at 4 °C and the supernatants were incubated with 100–200 µL KPBS prewashed Anti‐HA beads (Anti‐HA Affinity Gel) on a gentle shaker for 3 min. The immunoprecipitated were gently washed 3 times with KPBS on ice, and eluted with 2× loading sample buffer (4% SDS, 20% glycerol, 10% β‐ME, 0.004% bromophenol blue and 0.125 M Tris‐HCl, pH 6.8). The samples were analyzed by western blotting.

### Statistical Analysis

Data were presented as mean ± standard deviation (SD) of all samples analyzed in multiple experiments (that is, with more than three biological replicates). Statistical significance was determined using unpaired two‐tailed Student's *t* test for samples with one variable, or two‐way ANOVA for samples with more than one variable or more than two groups. For imaging analyses, two‐tier tests were used to first combine technical replicates and then evaluate biological replicates. Statistical analyses were performed with R packages version 4.3.2. Statistical significance was defined as a *p*‐value less than 0.05, with significance levels indicated as *p* < 0.05 (*), *p* < 0.001 (***).

## Conflict of Interest

The authors declare no conflict of interest.

## Supporting information



Supporting Information

## Data Availability

The data that support the findings of this study are available from the corresponding author upon reasonable request.
