# Supplementary material for: Gal3‐CaN‐Smurf1 Complex Sequestrates FLCN‐FNIPs to Facilitate TFEB Activation in Response to Endomembrane Damage
Source: Adv Sci (Weinh). 2025 Sep 17;12(40):e13241. doi: 10.1002/advs.202413241 (PMC12561182; doi:10.1002/advs.202413241)
Supplement: Supplementary file 1 — Supporting Information [file ADVS-12-e13241-s001.pdf]

Supplementary Figure 1: Smurf1 promotes the disassociation of RagC from TFEB

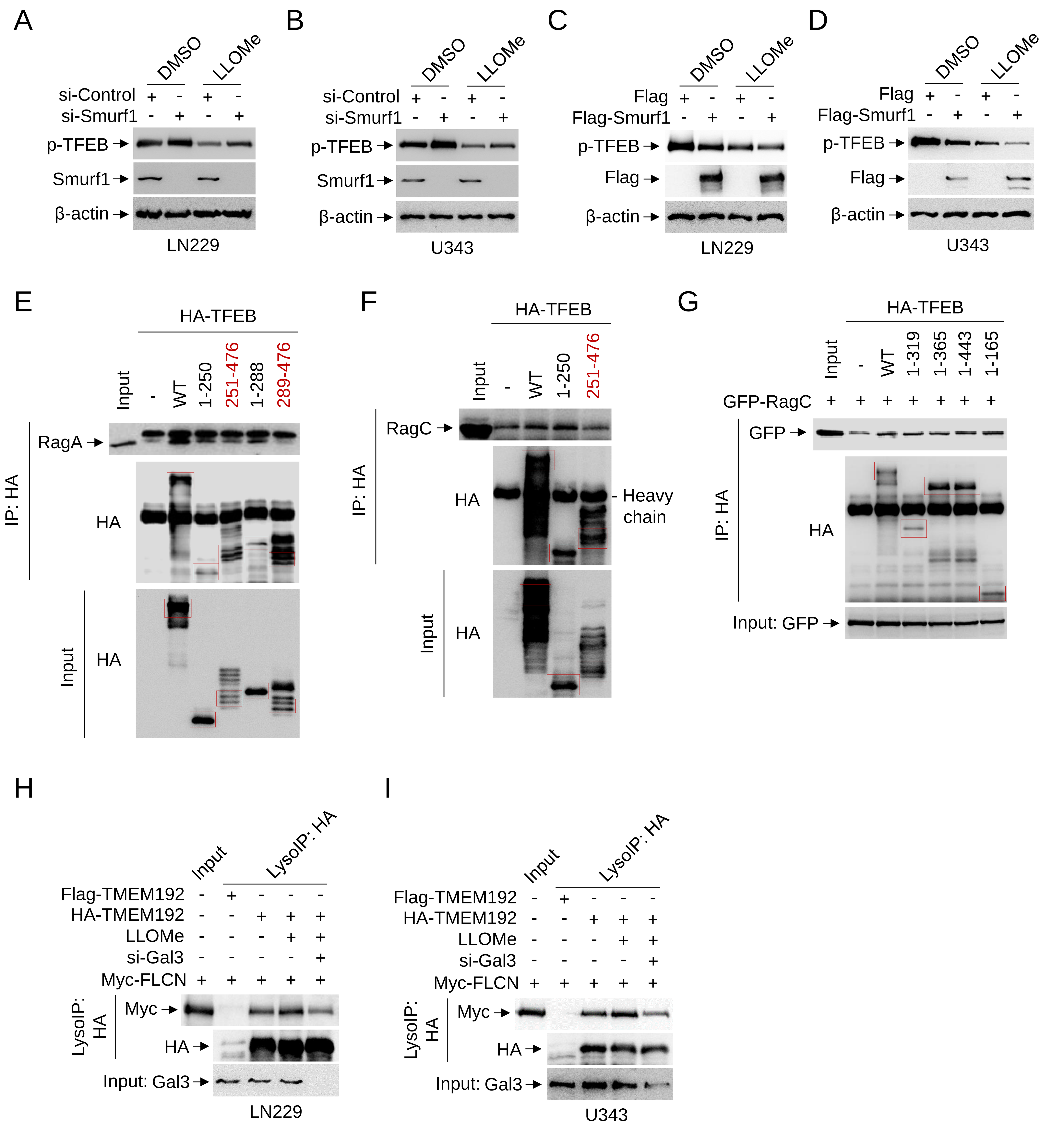

Supplementary Figure 1: Smurf1 promotes the disassociation of RagC from TFEB
(A, B) LN229 and U343 cells were transfected with Smurf1 or scramble siRNA oligos for 72 h and then treated with LLOMe (1 mM, 2 h) or equal volume DMSO. Cells were subjected to western blotting using p-TFEB, Smurf1 and β-actin antibodies. (C, D) LN229 and U343 cells were transfected with Flag or Flag-Smurf1 for 24 h, and then were treated with LLOMe (1 mM, 2 h) or equal volume DMSO. Cells were subjected to western blotting using p-TFEB, Flag and β-actin antibodies. (E-G) Co-IP analysis of the interaction between HA-TFEB constructs and endogenous RagA (E) or endogenous RagC (F) or overexpressed GFP-RagC (G) in HEK293 cells. (H, I) LN229 cells and U343 cells were transfected with Gal3 or scramble siRNA oligos for 72 h and transfected with Flag-TMEM192 or HA-TMEM192 and Myc-FLCN for another 24 h. Cells were treated with LLOMe (1 mM, 2 h) or equal volume DMSO and subjected to LysoIP with HA for immunoblotting with antibodies against HA, Myc and Gal3. Data are representative of three independent experiments with three biological replicates.

Supplementary Figure 2: Smurf1 directly interacts with and ubiquitylates FLCN

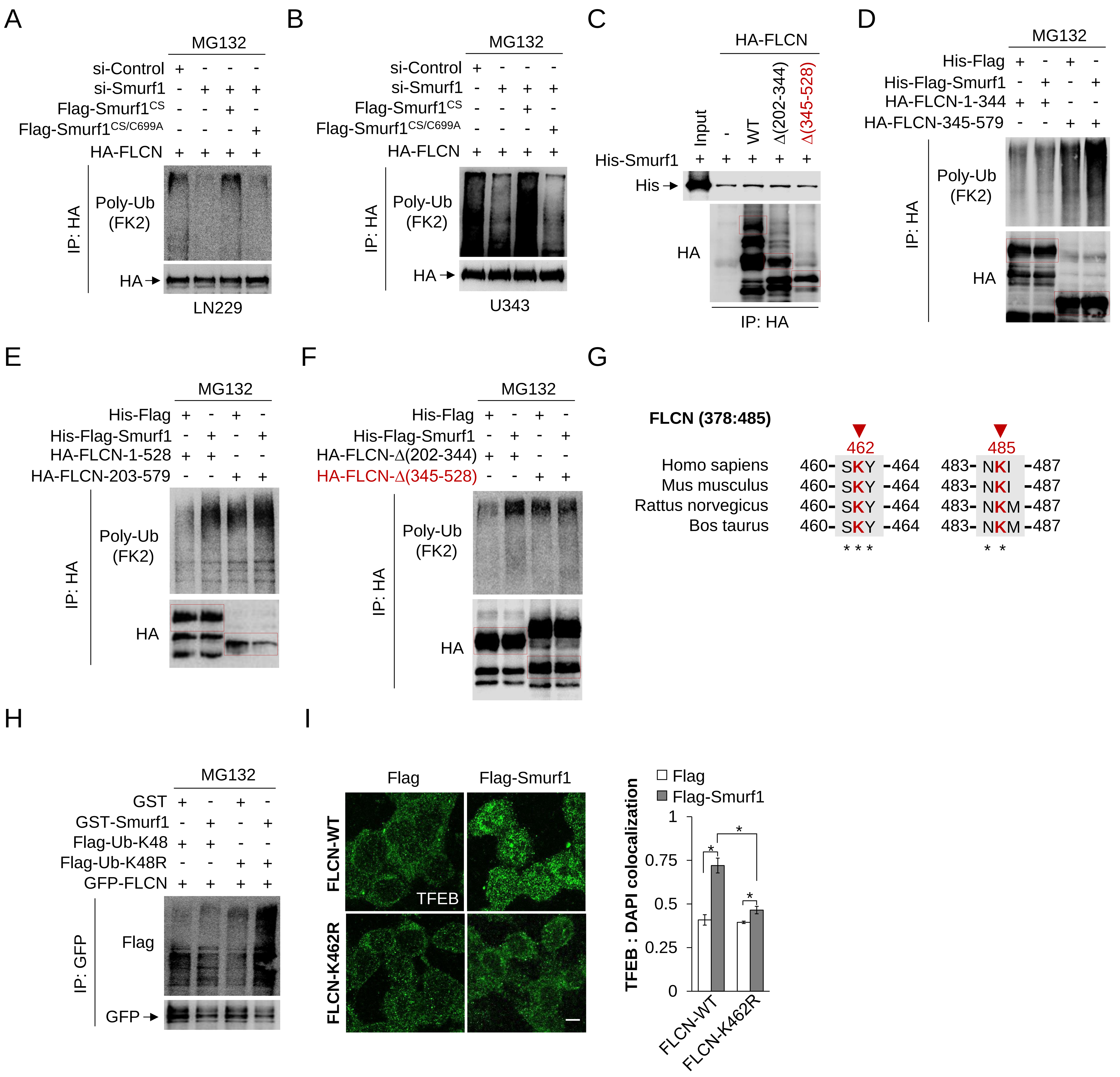

Supplementary Figure 2: Smurf1 directly interacts with and ubiquitylates FLCN

(A, B) LN229 and U343 cells were transfected with Smurf1 or scramble siRNA oligos for 48 h and transfected with Flag-Smurf1-CS (Codon Switch: resistance to si-Smurf1) or Flag-Smurf1-CS/C699A and HA-FLCN for another 12 h. Cells were then treated with MG132 (10 μM) for 12 h. Cell lysates were then IP with HA for immunoblotting with antibodies against Ub and HA. (C) Co-IP analysis of the interaction between HA-FLCN constructs expressed in HEK293 cells and His-Smurf1 purified from E. coli. (D-F) HEK293 cells were transfected with HA-FLCN constructs for 12 h and treated with MG132 (10 μM) for another 12 h. Cell lysates were then incubated with or without His-Flag-Smurf1 and IP with HA for immunoblotting with antibodies against Ub and HA. (G) Query the gene database for sequences of FLCN from several eukaryotes and analyze the relatively conserved lysine residues in the sequences. (H) HEK293 cells were transfected with GFP-FLCN and Flag-Ub-K48 or Flag-Ub-K48R for 12 h and treated with MG132 (10 μM) for another 12 h. Cell lysates were then incubated with or without GST-Smurf1 and IP with GFP for immunoblotting with antibodies against Flag and GFP. (I) HEK293 cells were transfected with FLCN siRNA oligos for 48 h and transfected with Flag-Smurf1 and HA-FLCN-WT or HA-FLCN-K462R for another 12 h before fixation and staining with TFEB antibody (G). The percentage of cells with TFEB in nucleus is shown in (H). Scale bar: 5 μm. n ≥ 50 cells per group. Data are representative of three independent experiments with three biological replicates. Results are presented as mean ± SD, *$p < 0.05$ by two-sided Student's t-test.

Supplementary Figure 3: Smurf1 indirectly interacts with FNIPs

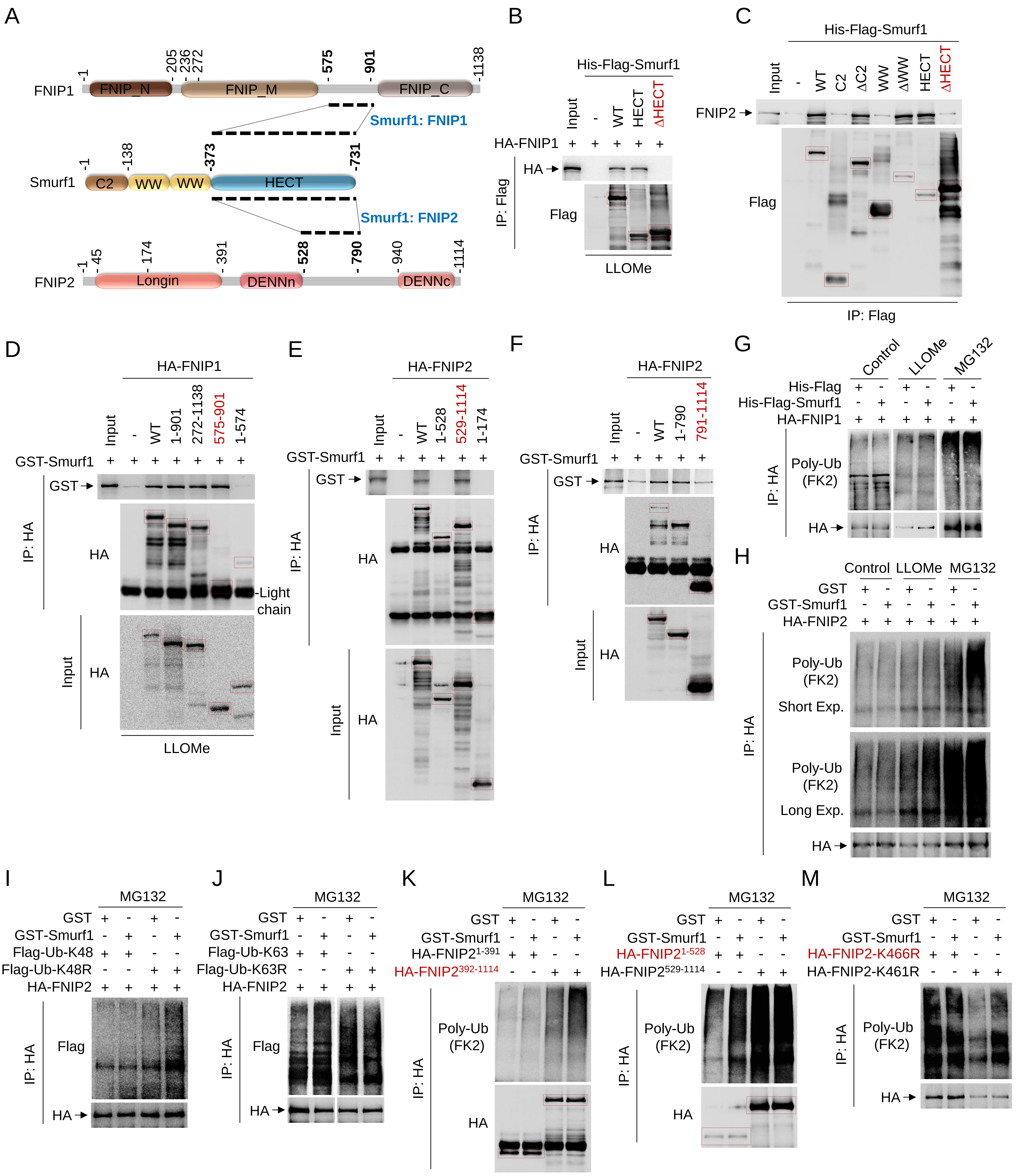

Supplementary Figure 3: Smurf1 indirectly interacts with FNIPs

(A) Schematic diagram of mapping the interaction between Smurf1 and FNIP1 or FNIP2. (B) Co-IP analysis of the interaction between HA-FNIP1 expressed in HEK293 cells and His-Flag-Smurf1 constructs purified from E. coli. (C) Co-IP analysis of the interaction between His-Flag-Smurf1 constructs purified from E. coli and endogenous FNIP2 in HEK293 cells. (D-F) Co-IP analysis of the interaction between HA-FNIP1 (D) or HA-FNIP2 (E, F) constructs expressed in HEK293 cells and GST-Smurf1 purified from E. coli.

(G, H) HEK293 cells were transfected with HA-FNIP1 (G) or HA-FNIP2 (H) for 24 h and treated with MG132 (10 μM, 12 h) or LLOMe (1 mM, 2 h). Cell lysates were then incubated with or without His-Flag-Smurf1 (G) or GST-Smurf1 (H) and IP with HA for immunoblotting with antibodies against Ub and HA. (I, J) HEK293 cells were transfected with HA-FNIP2 and Flag-Ub-K48 or Flag-Ub-K48R (I) and Flag-Ub-K63 or Flag-Ub-K63R (J) for 12 h and treated with MG132 (10 μM) for another 12 h. Cell lysates were then incubated with or without GST-Smurf1 and IP with HA for immunoblotting with antibodies against Flag and HA. (K-M) HEK293 cells were transfected with HA-FNIP2 constructs for 12 h and treated with MG132 (10 μM) for another 12 h. Cell lysates were then incubated with or without GST-Smurf1 and IP with HA for immunoblotting with antibodies against Ub and HA. Data are representative of three independent experiments with three biological replicates.

Supplementary Figure 3-1: Smurf1 indirectly interacts with FNIPs

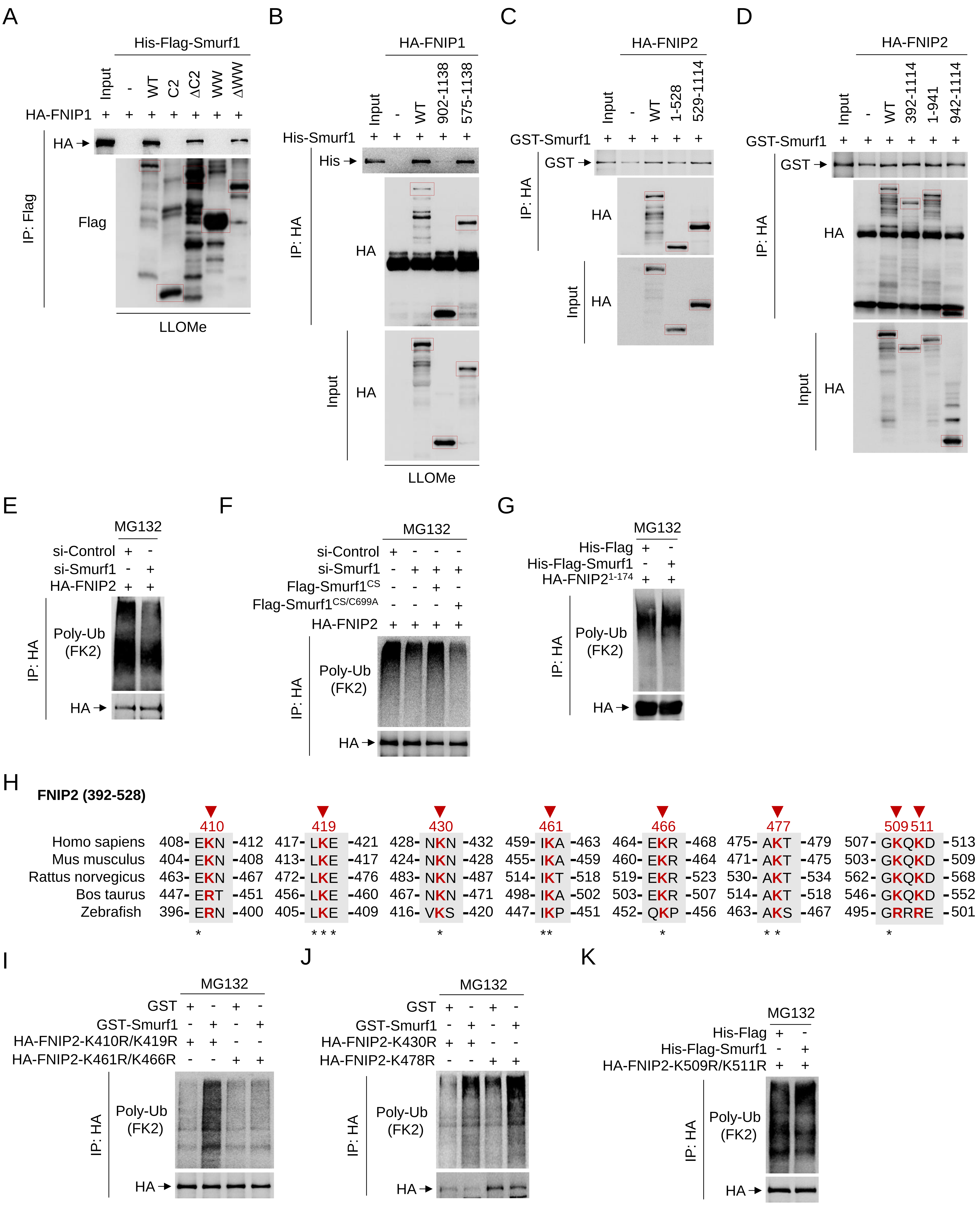

Supplementary Figure 3-1: Smurf1 indirectly interacts with FNIPs

(A) Co-IP analysis of the interaction between HA-FNIP1 expressed in HEK293 cells and His-Flag-Smurf1 constructs purified from E. coli. (B) Co-IP analysis of the interaction between HA-FNIP1 constructs expressed in HEK293 cells and His-Smurf1 purified from E. coli. (C, D) Co-IP analysis of the interaction between HA-FNIP2 constructs expressed in HEK293 cells and GST-Smurf1 purified from E. coli. (E) HEK293 cells were transfected with Smurf1 or scramble siRNA oligos for 48 h and transfected with HA-FNIP2 for another 12 h, and then were treated with MG132 (10 μM, 12 h). Cell lysates were IP with HA for immunoblotting with antibodies against Ub and HA.

(F) HEK293 cells were transfected with Smurf1 or scramble siRNA oligos for 48 h and transfected with HA-FNIP2 and Flag-Smurf1-CS or Flag-Smurf1-CS/C699A for another 12 h, and then were treated with MG132 (10 μM, 12 h). Cell lysates were IP with HA for immunoblotting with antibodies against Ub and HA. (G) HEK293 cells were transfected with HA-FNIP2 (1-174) for 12 h and then were treated with MG132 (10 μM, 12 h). Cell lysates were then incubated with or without His-Flag-Smurf1 and IP with HA for immunoblotting with antibodies against Ub and HA. (H) Query the gene database for sequences of FNIP2 from several eukaryotes and analyze the relatively conserved lysine residues in the sequences. (I-K) HEK293 cells were transfected with HA-FNIP2 constructs for 12 h and then were treated with MG132 (10 μM, 12 h). Cell lysates were then incubated with or without GST-Smurf1 or His-Flag-Smurf1 and IP with HA for immunoblotting with antibodies against Ub and HA. Data are representative of three independent experiments with three biological replicates.

Supplementary Figure 4: Gal3-CaN-Smurf1 complex interacts with FLCN-FNIPs

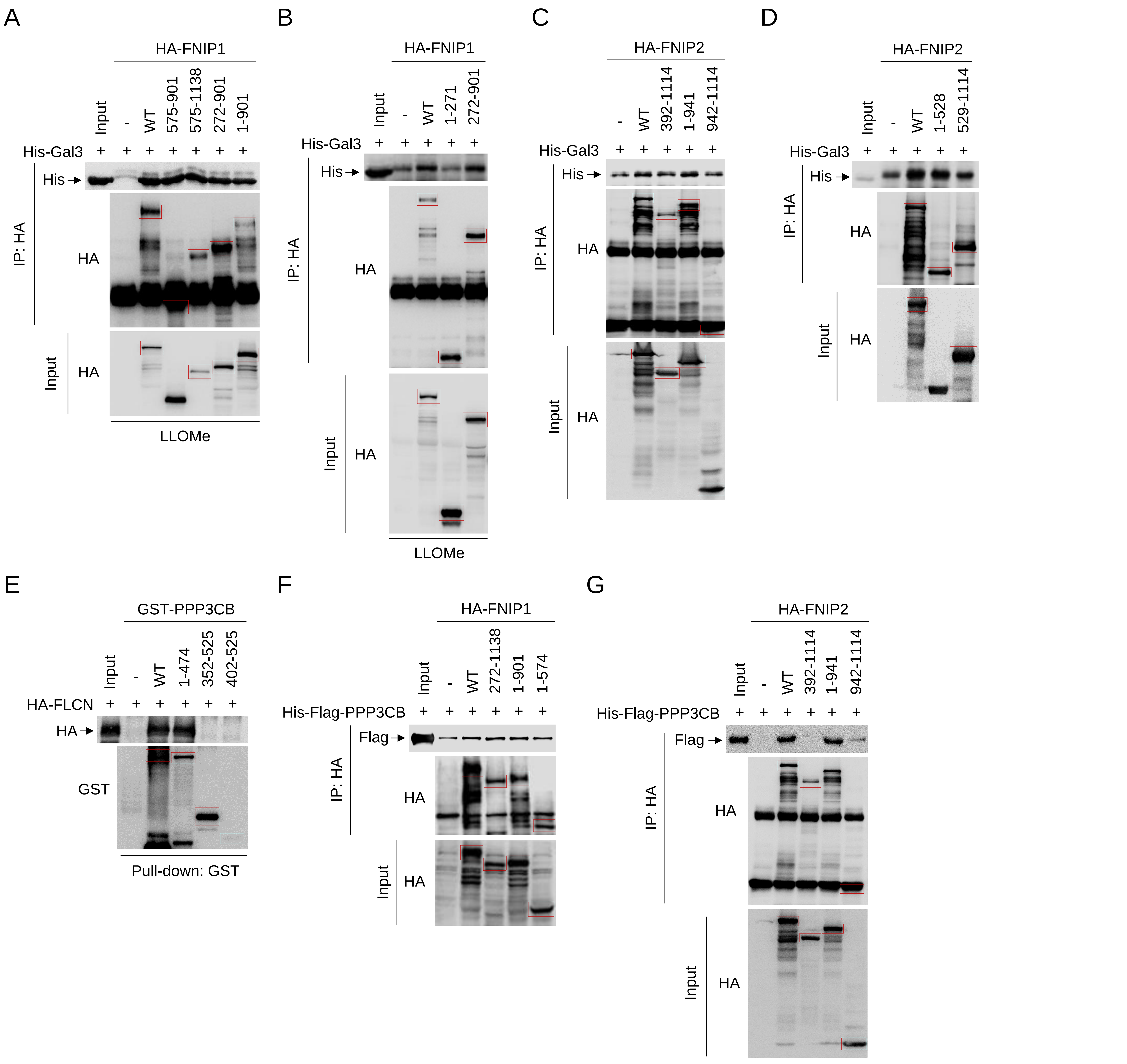

Supplementary Figure 4: Gal3-CaN-Smurf1 complex interacts with FLCN-FNIPs
(A-D) Co-IP analysis of the interaction between HA-FNIP1 (A, B) or HA-FNIP2 (C, D) constructs expressed in HEK293 cells treated with or without LLOMe (1 mM, 2 h) and His-Gal3 purified from E. coli. (E) GST Pull-down analysis of the interaction between HA-FLCN expressed in HEK293 cells and GST-PPP3CB constructs purified from E. coli. (F, G) Co-IP analysis of the interaction between HA-FNIP1 (F) or HA-FNIP2 (G) constructs expressed in HEK293 cells and His-Flag-PPP3CB purified from E. coli. Data are representative of three independent experiments with three biological replicates.

Supplementary Figure 5: The Gal3-CaN-Smurf1 complex facilitates sequestration of FLCN-FNIPs

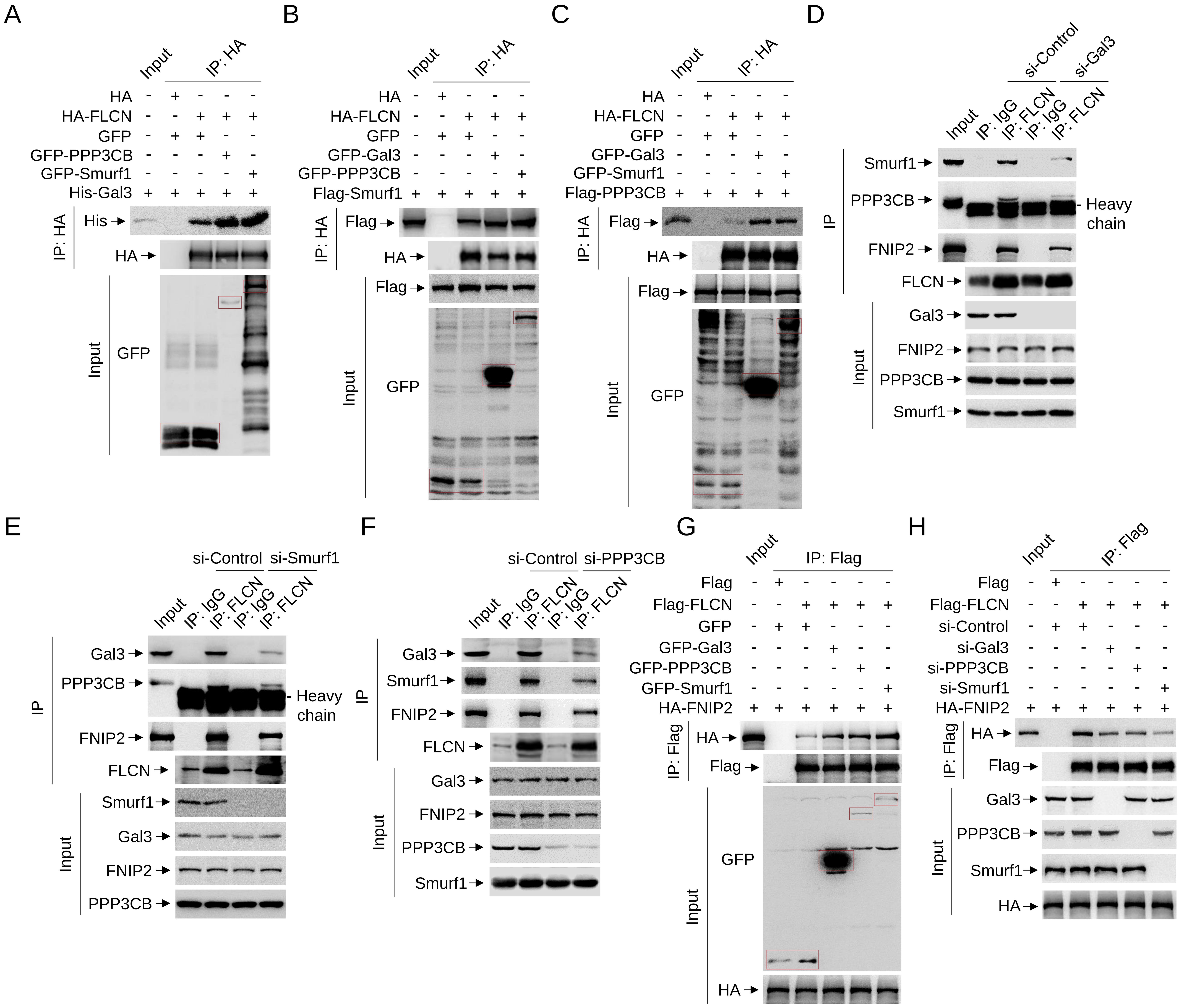

Supplementary Figure 5: The Gal3-CaN-Smurf1 complex facilitates sequestration of FLCN-FNIPs

(A) Co-IP analysis of the interaction between HA-FLCN expressed in HEK293 cells and His-Gal3 purified from E. coli with or without FLCN. (B, C) Co-IP analysis of the interaction between HA-FLCN and Flag-Smurf1 (B) or Flag-PPP3CB (C) expressed in HEK293 cells. (D-F) Co-IP analysis of the interaction between endogenous FLCN and indicated endogenous proteins in HEK293 cells. (G, H) Co-IP analysis of the interaction between Flag-FLCN and HA-FNIP2 expressed in HEK293 cells. Data are representative of three independent experiments with three biological replicates.

Supplementary Figure 6: Smurf1 interacts with and ubiquitylates TFEB

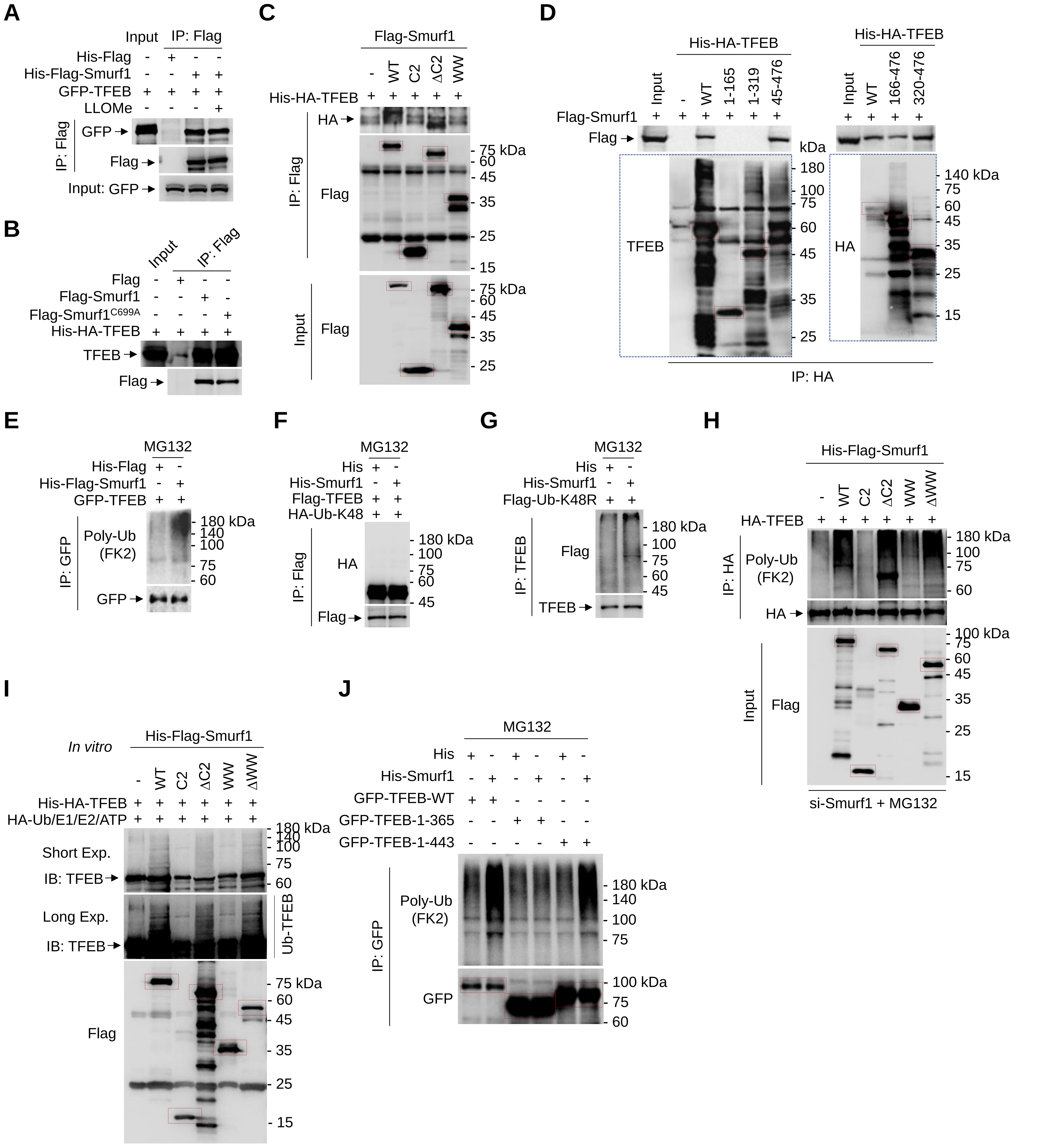

Supplementary Figure 6: Smurf1 interacts with and ubiquitylates TFEB

(A) HEK293 cells were transfected with GFP-TFEB for 22 h and then treated with or without LLOMe (1 mM, 2 h). Co-IP analysis of the interaction between GFP-TFEB expressed in HEK293 cells and His-Flag-Smurf1 purified from E. coli. (B, C) Co-IP analysis of the interaction between Flag-Smurf1 constructs expressed in HEK293 cells and His-HA-TFEB purified from E. coli. (D) Co-IP analysis of the interaction between Flag-Smurf1 expressed in HEK293 cells and His-HA-TFEB constructs purified from E. coli. (E) HEK293 cells were transfected with GFP-TFEB and treated with MG132 (10 μM, 12 h). Cell lysates were then incubated with or without His-Flag-Smurf1 and IP with GFP for immunoblotting with antibodies against Ub and GFP. (F) HEK293 cells were transfected with Flag-TFEB and HA-Ub-K48 and treated with MG132 (10 μM, 12 h). Cell lysates were then incubated with or without His-Smurf1 and IP with Flag for immunoblotting with antibodies against HA and Flag. (G) HEK293 cells were transfected with Flag-Ub-K48R and treated with MG132 (10 μM, 12 h). Cell lysates were then incubated with or without His-Smurf1 and IP with TFEB for immunoblotting with antibodies against TFEB and Flag.

(H) HEK293 cells were transfected with si-Smurf1 and HA-TFEB, and then treated with MG132 (10 μM, 12 h). Cell lysates were incubated with or without His-Flag-Smurf1 constructs and IP with HA for immunoblotting with antibodies against HA, Flag and Ub. (I) His-Flag-Smurf1 constructs (purified from E. coli) were IP with Flag antibody, and then added purified His-HA-TFEB, HA-Ub, E1, E2 (UbcH5c) and ATP to perform in vitro ubiquitination assay. Ubiquitinated TFEB detected by immunoblotting against TFEB antibody. (J) HEK293 cells were transfected with GFP-TFEB constructs and treated with MG132 (10 μM, 12 h). Cell lysates were incubated with or without His-Smurf1 and IP with GFP for immunoblotting with antibodies against GFP and Ub. Data are representative of three independent experiments with three biological replicates.
